# Gradual change of cortical representations with growing visual expertise for synthetic shapes

**DOI:** 10.1162/imag_a_00255

**Published:** 2024-08-06

**Authors:** Ehsan Kakaei, Jochen Braun

**Affiliations:** European Structural and Investment Funds Graduate School on Analysis, Imaging, and Modelling of Neuronal and Inflammatory Processes, Otto-von-Guericke University, Magdeburg, Germany; Institute of Biology, Otto-von-Guericke University, Magdeburg, Germany; Center for Behavioral Brain Sciences, Otto-von-Guericke University, Magdeburg, Germany

**Keywords:** object recognition, visual expertise, functional imaging, representational similarity, and multi-voxel activity

## Abstract

*Objective*: Visual expertise for particular categories of objects (e.g., mushrooms, birds, flowers, minerals, and so on) is known to enhance cortical responses in parts of the ventral occipitotemporal cortex. How is such additional expertise integrated into the prior cortical representation of life-long visual experience? To address this question, we presented synthetic visual objects rotating in three dimensions and recorded multivariate BOLD responses as initially unfamiliar objects gradually became familiar.

*Main results*: An analysis of pairwise distances between multivariate BOLD responses (“representational similarity analysis,” RSA) revealed that visual objects were linearly discriminable in large parts of the ventral occipital cortex, including the primary visual cortex, as well as in certain parts of the parietal and frontal cortex. These cortical representations were present from the start, when objects were still unfamiliar, and even though objects were shown from different sides. As shapes became familiar with repeated viewing, the distribution of responses expanded to fill more of the available space. In contrast, the distribution of responses to novel shapes (which appeared only once) contracted and shifted to the margins of the available space.

*Conclusion*: Our results revealed cortical representations of object shape and gradual changes in these representations with learning and consolidation. The cortical representations of once-viewed shapes that remained novel diverged dramatically from repeatedly viewed shapes that became familiar. This disparity was evident in both the similarity and the diversity of multivariate BOLD responses.

## 1 Introduction

An essential aspect of visual object recognition is the processing of visual shapes. The neural substrate of shape processing includes the ventral visual pathway, which in humans extends over the ventral occipitotemporal cortex from the occipital pole to the lateral occipital cortex, fusiform gyrus, and beyond (reviewed byBi et al., 2016;Grill-Spector & Weiner, 2014;Kravitz et al., 2013;Weiner & Zilles, 2016). Functional imaging studies of ventral occipitotemporal cortex reveal intriguing functional anatomy, with responsiveness to specific object categories (e.g., faces, scenes, body parts) changing systematically over the cortical surface along several large-scale anatomical gradients (e.g., animate-inanimate, large-small, feature-whole, or perception-action;Freud et al., 2017;Grill-Spector & Weiner, 2014;Grill-Spector et al., 2004;Konkle & Oliva, 2012;Wurm & Caramazza, 2022;Yildirim et al., 2019).

Experience and learning improve object recognition performance, and also modify shape processing in the ventral occipitotemporal cortex. Indeed, functional imaging evidence shows that particular visual expertise—being able to identify and categorize visually similar objects of a particular kind—often entails moderate but anatomically distributed changes in the pre-existing responsiveness to shape (reviewed byBukach et al., 2006;de Beeck & Baker, 2010;Gauthier & Tarr, 2016;Harel et al., 2013). This has been established by comparing novices and experts for identifying particular categories of natural objects (e.g., birds, mushrooms, minerals, degraded images;Cetron et al., 2019;Connolly et al., 2012;Duyck et al., 2021;Freud et al., 2017;Martens et al., 2018;McGugin et al., 2012;Roth & Zohary, 2015), as well as by comparing observers before and after they have learned to categorize initially unfamiliar synthetic shapes (e.g., computer-generated “greebles,” “spikies,” or “ziggerins”;Brants et al., 2011;de Beeck et al., 2006;Gauthier et al., 1999;A. C.-N. Wong et al., 2009;Y. K. Wong et al., 2012;Yue et al., 2006).

Here, we map the cortical representation of synthetic visual objects and track gradual changes as initially unfamiliar objects become progressively familiar with learning. We wondered how pre-existing shape representations would accommodate and integrate novel synthetic objects. We further wondered whether representational changes would be specific to learned objects or extend also to other objects of the same kind. To explore these questions, we analyzed “representational similarity” of spatiotemporal BOLD patterns (Haxby, 2012;Kriegeskorte, Mur, Ruff, et al., 2008), which offers a potentially sensitive measure for the information encoded in neural activity and may also be related to similarity as perceived by human observers (Charest & Kriegeskorte, 2015;Collins & Behrmann, 2020;Nestor et al., 2016).

Most previous studies of visual expertise identified cortical sites associated with a particular object category by comparing BOLD activity either between novices and experts or before and after learning. We extend this work in three ways: firstly, by establishing representational distance at the level of object exemplars rather than object categories; secondly, by monitoring gradual changes as observers gain familiarity with object exemplars; and thirdly, by analyzing changes in the diversity of multivariate BOLD activity. Few previous studies have attempted to resolve shape representations in such detail (Brants et al., 2016;Duyck et al., 2021;Eger et al., 2008;Visconti di Oleggio Castello et al., 2021). To progress fine-grained analysis of representational geometry, we developed synthetic shapes for which visual expertise is acquired comparatively slowly (Kakaei et al., 2021) and took advantage of a numerically tractable method for linear discriminant analysis in O($10^{3}$)-dimensional multivariate activity (DLDA;Yu & Yang, 2001).

Our results showed view-invariant representations of shape over surprisingly extensive regions of the ventral occipitotemporal cortex, including the fusiform gyrus, lateral occipital areas, and primary visual cortex. Representational distances were high from the start, even before learning, suggesting that new visual expertise was accommodated and encoded within pre-existing representations. However, shapes that appeared repeatedly (and were memorized by observers) and shapes that appeared just once (and were ignored) diverged dramatically, in terms of their cortical representations, while visual expertise was being acquired and consolidated.

## 2 Methods

### 2.1 Observers and behavior

Eight healthy observers (4 female and 4 male; aged 25 to 32 years) took part in behavioral training (“sham experiment,” one session per observer), the functional imaging experiment (“main experiment,” six scanning sessions per observer), and a final behavioral assessment (two sessions). All observers were paid and gave informed consent. Ethical approval was granted under Chiffre 30/21 by the ethics committee of the Faculty of Medicine of the Otto-von-Guericke University, Magdeburg.

In both sham and main experiments, observers viewed sequences of 200 recurring and non-recurring objects (see below andFig. 1A) and attempted to classify each object as “familiar” or “novel” (by pressing the appropriate button). Over the course of multiple sessions, observers gradually became familiar with recurring objects and thus became able to distinguish them from non-recurring objects. Objects of the sham experiment were two-dimensional shapes, whereas objects of the main experiment were rotating, three-dimensional shapes (see below andFig. 1A).

**Fig. 1.**
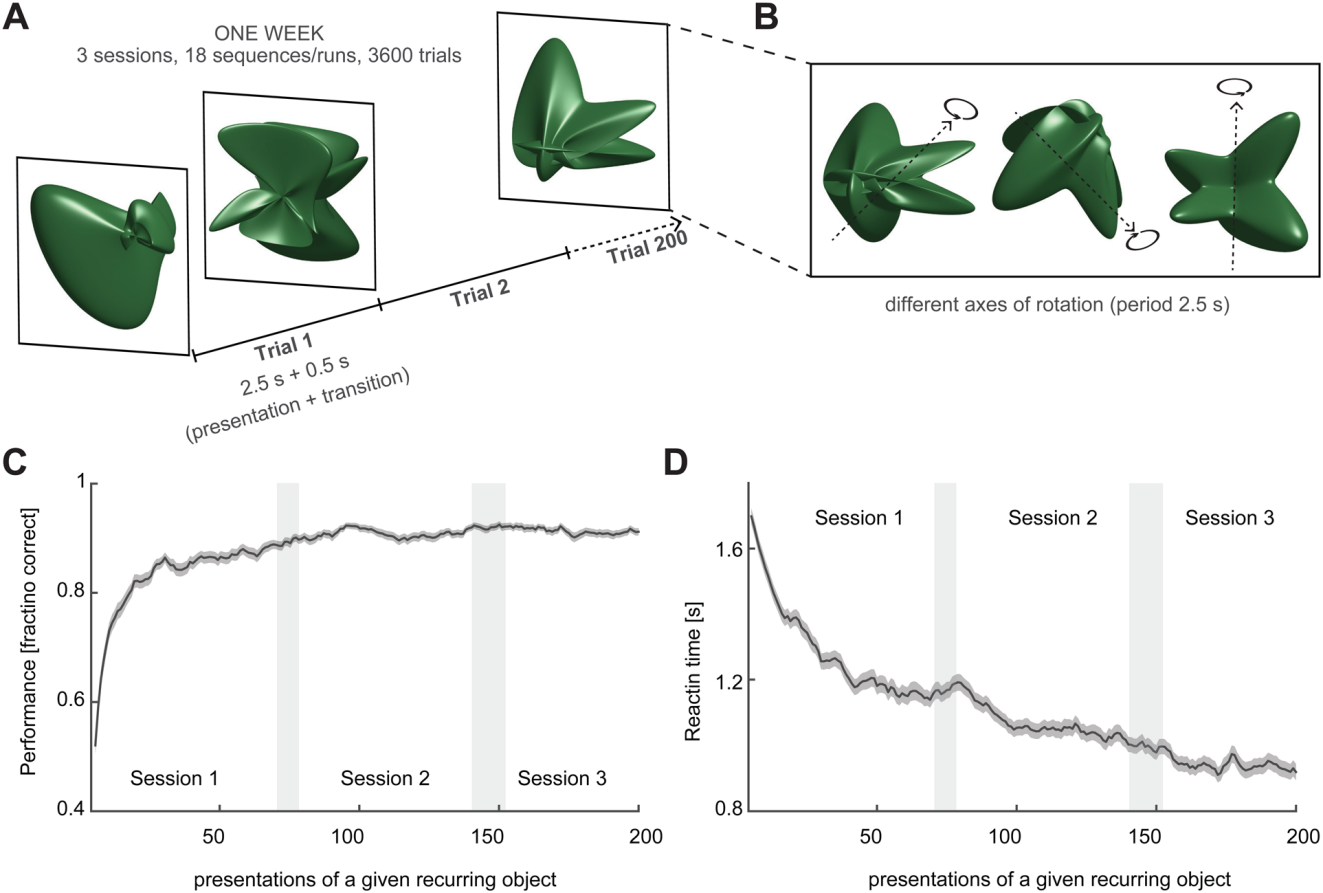
Experimental paradigm. (A) Complex objects were shown for 2.5 s each, separated by 0.5 s transition, in sequences of 200 presentations, with a total duration of 600 s. Over 1 week, observers participated in 3 sessions, viewing 6 sequences during each session (18 sequences in total). Fifteen objects appeared many times each (“recurring objects”), while other objects appeared exactly once (“non-recurring objects”). Observers were required to categorize each object as either “familiar” or “unfamiliar” (by button press). (B) Objects appeared randomly rotated and revolved for one full turn (clockwise or counter-clockwise about variable axes in the frontal plane, inclination of$0^{\circ }$,$45^{\circ }$, or$-45^{\circ }$). (C) Over the course of the week, as observers became familiar with recurring objects, classification performance improved. Here, performance (average and S.E.M.) is shown as a function of the number of presentations for 15 recurring objects, 8 observers, and 2 conditions. The relation between presentations and sessions was probabilistic (indicated by gray shading). (D) Reaction time (average and S.E.M.) as a function of presentation number. With increasing familiarity, reaction times decrease by 50% (from1.7sto0.9s) and become considerably shorter than the presentation time.

The main experiment extended over 3 successive weeks, with three sessions on separate days of both the 1st and 3rd week (no sessions took place in the 2nd week). The experiments of the 1st and 3rd week differed in four aspects: sequence type (structured or unstructured), the set of recurring objects, object color (red or blue), and responding hand (left or right). All aspects were counterbalanced across observers.

After the three scanning sessions of a week, observers participated in an additional behavioral session to confirm that they had in fact become familiar with every recurring object. Specifically, they performed a spatial search task in which they pointed out recurring target objects among non-recurring distractor objects (Kakaei et al., 2021). In addition, observers were offered the opportunity to voice anything they might have noticed about the experiment.

### 2.2 Experimental paradigm

Complex three-dimensional objects were computer-generated and presented as described previously (Kakaei et al., 2021). A movie can be viewed under thisLINK. All objects were highly characteristic and dissimilar from each other as confirmed computationally in terms of vector distances between depth maps (Kakaei et al., 2021). Objects were presented every3s, with2.5sviewing and0.5stransition time (Fig. 1A). Objects were shown from all sides and, after appearing at an arbitrary angle, revolved smoothly for one full turn (period2.5s, frequency0.4Hz, angular frequency$144^{\circ }/s$) about one of several axes in the frontal plane ($-45^{\circ }$,$0^{\circ }$,$45^{\circ }$, clockwise or counter-clockwise). Axes and directions were counterbalanced for each object, and initial viewing angles were chosen randomly (Fig. 1B). All stimuli were generated with MATLAB (The MathWorks, Inc.), presented with the psychophysics toolbox (Brainard, 1997), and viewed in a mirror mounted to the MR head coil (screen resolution960×720pixels, frame rate60Hz, subtending approximately$8^{\circ }\times 6^{\circ }$of visual angle, average luminance$50Cd/m^{2}$, background luminance$5Cd/m^{2}$). Observers responded with the right or left index finger on an MR-safe response box.

Fifteen objects recurred many times during three sessions (“recurring” objects), whereas other objects appeared exactly once (“non-recurring” or “singular” objects). As mentioned, observers classified every object as either “familiar” or “unfamiliar” by pressing a button during its presentation. Over the course of three sessions, all observers gradually became familiar with the “recurring objects” (see below). The average time-course of learning, as established by a simplified signal detection and reaction-time (RT) analysis, is shown inFigure 1C.

Every session comprised six sequences (“runs”), each lasting600sand presenting180“recurring” and20“non-recurring” objects (200objects in total). As there were 15 different recurrent objects, each such object was seen12±1.9times during every sequence. Over the three sessions (or 18 sequences), each recurring object appeared at least190times each (mean±S.D.:216±9), whereas non-recurring objects appeared only once. Altogether, there were3,240presentations of recurring objects (3×6×180) and360presentations of non-recurring objects (3×6×180).

Presentation sequences started with a random recurring object and continued randomly to one of the possible next objects, with neither immediate repetitions (X→X) nor direct returns (X→Y→X) being allowed. Sequences comprised200objects, of which180were recurring and20objects non-recurring and were interspersed at random intervals. Object sequences were post-selected such as to counterbalance the number of appearances of every recurring object in every session.

All observers performed the experiment twice in the scanner, once during the 1st week and again during the 3rd week of the main experiment (so that 8 observers provided 16 data sets). As mentioned, the 2 weeks differed in terms of the recurring objects and the presentation sequence. “Structured” sequences exhibited predictive sequential dependencies (3 possible recurring next objects), whereas “unstructured” sequences did not (14 possible recurring next objects, seeKakaei et al., 2021for details). As a result, the repetition latency (i.e., the latency of successive presentations of the same object) was5.5±15(median and S.D.) for “structured” and10.5±11for “unstructured” sequences. Further aspects and effects of sequence structure are reported and discussed in detail in a companion paper.

To verify that recurring objects had become familiar to observers, every observer performed 60 trials of a spatial search task with 3 recurring and 9 non-recurring objects. The 12 objects were positioned randomly in a3×4array and were presented for 30 s while rotating in three dimensions (as in the main experiment). After each presentation, observers indicated the recurring object positions with the computer mouse. Performance was consistently above 95% correct.

### 2.3 MRI acquisition

All magnetic-resonance images were acquired on a 3T Siemens Prisma scanner with a 64-channel head coil. Structural images were T1-weighted sequences (**MPRAGE**TR = 2,500 ms, TE = 2.82 ms, TI = 1,100 ms,$7^{\circ }$flip angle, isotropic resolution1×1×1mmand matrix size of256×256×192). Functional images were T2*-weighted sequences (TR = 1,000 ms, TE = 30 ms,$65^{\circ }$flip angle, resolution of3×3×3.6mmand matrix size of72×72×36). Field maps were obtained by gradient dual-echo sequences (TR = 720 ms, TE1 = 4.92 ms, TE2 = 7.38 ms, resolution of1.594×1.594×2mmand matrix size of138×138×72).

### 2.4 fMRI pre-processing

Our approach to fMRI analysis was influenced by recent advances in comparing uni- and multivariate responses of corresponding voxels between different observers (e.g.,Kumar et al., 2022;Nastase et al., 2019). The*local*correlation structure of voxel response, which is similar in different observers, provided the basis for our functional parcellation (Dornas & Braun, 2018). The parcellation obviated “searchlight” strategies by defining for all observers corresponding brain “parcels” with corresponding episodes of high-dimensional (O(1000)) multivariate activity.

The fMRI pre-processing procedure was similar to that published previously (Dornas & Braun, 2018). First, DICOM files were converted into NIFTI format using MRICRON (MRICRON Toolbox, Maryland, USA, NIH). Then, brain tissues were extracted and segmented using BET (Smith, 2002) and FAST (Zhang et al., 2001). Field map correction, head motion correction, spatial smoothing, high-pass temporal filtering, and registration to structural and standard images were performed with the MELODIC package of FSL (Beckmann & Smith, 2004).

Field map correction and registration to structural image were carried out using Boundary-Based Registration (BBR;Greve & Fischl, 2009). MELODIC uses MCFLIRT (Jenkinson et al., 2002) to correct for head motion. Spatial smoothing was performed with SUSAN (Smith & Brady, 1997), with full width at half maximum set at FWHM=5mm. To remove low-frequency artifacts, we applied a high-pass filter of the cut-off frequencyf=0.01Hz, that is, oscillations/events with periods of more than100s were removed. To register the structural image to Montreal MNI152 standard space with isotropic2mmvoxel size, we used FLIRT (FMRIB’s Linear Image Registration Tool;Jenkinson & Smith, 2001;Jenkinson et al., 2002) with 12 degrees of freedom (DOF) and FNIRT (FMRIB’s Nonlinear Image Registration Tool) to apply the non-linear registration. To further reduce artifacts arising from head motion, we applied despiking with a threshold ofλ=100using BrainWavelet toolbox (Patel et al., 2014). Later, we regressed out the mean CSF activity as well as 12 DOF translation and rotation factors predicted by a motion correction algorithm (MCFLIRT). Afterward, the time series of each voxel was detrended linearly and whitened (with Matlab functions “detrend” and “zscore”).

Finally, the160,099voxels of MNI152 space were grouped into758functional parcels according to the MD758 atlas (Dornas & Braun, 2018). Each functional parcel is associated with an anatomically labeled region of the AAL atlas (Tzourio-Mazoyer et al., 2002) and comprises approximately200voxels or approximately$1.7cm^{\text{3}}$of gray matter volume (212±70voxels, range45to462voxels). Parcels were defined for a small population of observers such as to maximize signal covariance*within*and minimize covariance*between*parcels in the resting state. In contrast to other parcellation schemes, this was based exclusively on the (typically strong) functional correlations within each anatomical region and disregarded the (typically weak) correlations between different anatomical regions. The MD758 parcellation offers superior cluster quality, correlational structure, sparseness, and consistency with fiber tracking, compared to other parcellation schemes of similar resolution (Albers et al., 2021;Dornas & Braun, 2018).

### 2.5 fMRI data analysis

**Fig. 2.**
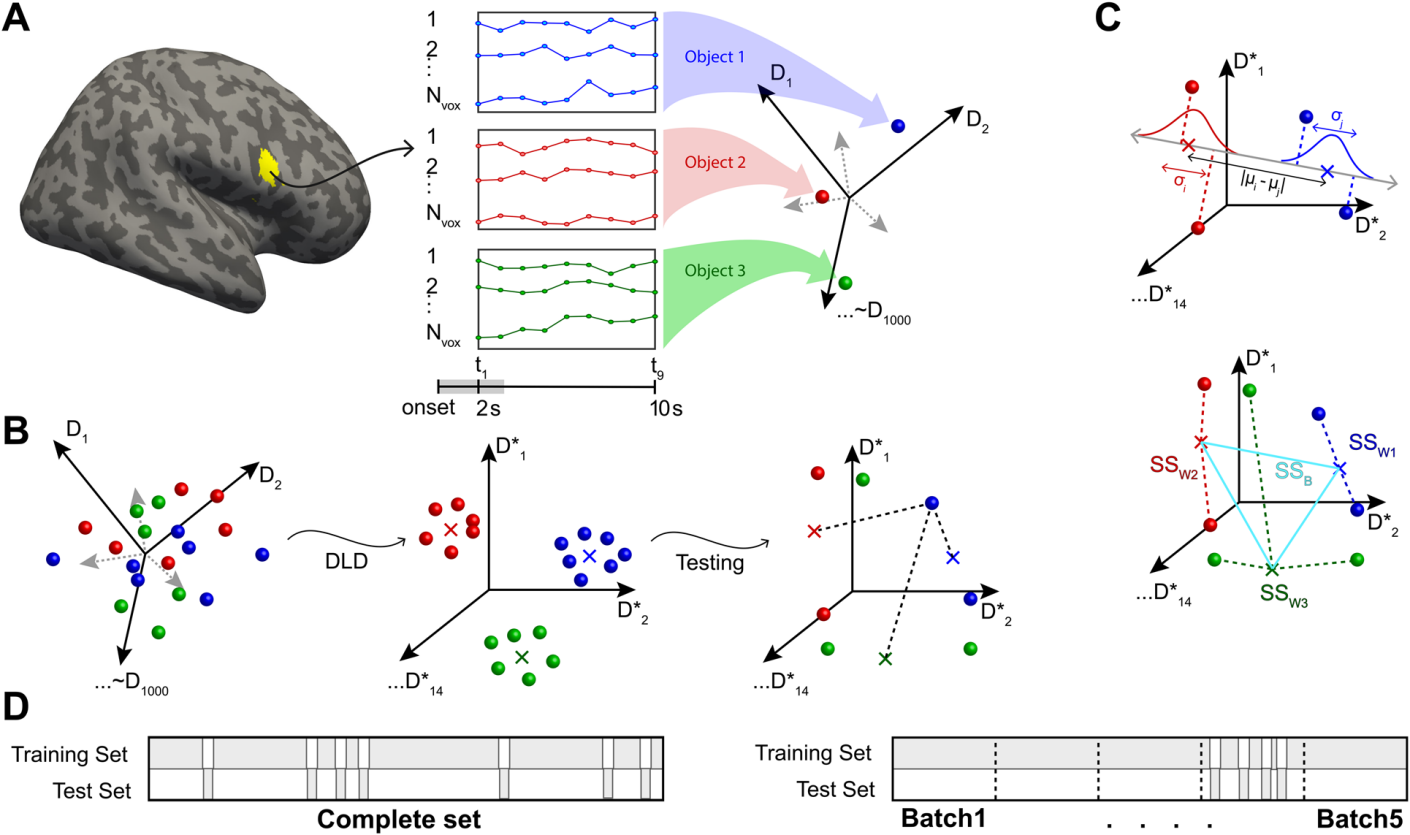
Analysis of fMRI activity with direct linear discriminant analysis, or DLDA. For each functional parcel, DLDA identified the14-dimensional space that optimally discriminated the15classes of activity patterns associated with15*recurring*objects. Other activity patterns, such as those associated with*nonrecurring*objects, were also analyzed in this space. (A) For a given parcel with$N_{vox}$voxels (e.g., yellow region Frontal-Inf-R-8), activity was recorded over9sduring and following object presentation (2to11safter onset). Each such activity pattern corresponds to a point (or vector) in a$9\cdot N_{vox}$-dimensional space (right). Here, activity patterns associated with three object presentations are represented schematically (red, green, and blue spheres). (B) To cross-validate discriminability, recurrent object presentations were divided randomly into a training set (90%) and a test set (10%). From the training set, the DLD subspaceSwas established. Here, exemplars (solid spheres) and class centroids (crosses) are represented schematically. Next, the projections into this space of test set patterns were compared to class centroids. (C) Projection onto the line connecting class centroidsiandjrevealed the pairwise discriminability/dissimilarity$\delta_{i,j}$of object classesiandj(top), and the distances to class centroids yielded the within-class and between-class variance of representations,$SS_{W}$and$SS_{B}$, and the associated variance ratio$F=SS_{B}/SS_{W}$(bottom). Additionally, a matrix of (mis-)classification probabilitiesP(reportedi|truej)(a.k.a confusion matrix) could be obtained (not shown). (D) To assess object representation generally, test presentations were drawn randomly from the complete set of object presentations (left). To assess changes over the duration of the experiment, the set of presentations was divided into five successive “batches” and test presentations were drawn from one of these batches (bottom). In either case, the training set comprised all remaining presentations (i.e., the complement of the test set).

To study the neural representation of objects, we extracted the multivoxel activity pattern at$N_{t}=9$time points following object onset. In a functional parcel with$N_{vox}$voxels, this response pattern constituted a point (or vector) in an$N_{dim}$-dimensional space, where$N_{dim}=N_{t}\cdot N_{vox}$(Fig. 2A). To identify parcels with significant selectivity for individual recurring objects, we employed a representational similarity analysis (RSA;Kriegeskorte, Mur, & Bandettini, 2008) (Fig. 2B). This analysis uses the standardized Euclidean (Mahalanobis) distance between responses in a high-dimensional space to examine the separability of neural object representations as a function of learning, or object type (recurring or non-recurring), or both. Over all 758 parcels, response dimensionality was$N_{dim}=1,911\pm 634$(mean and standard-deviation), with a range from405(Calcarine-L 329, with45voxels) to4,113(Postcentral-R-484, with457voxels).

Our approach to RSA differed from previous work in some respects. Firstly, we analyzed high-dimensional*spatiotemporal*patterns of BOLD activity (200voxels×9s, or O($10^{3}$) dimensions) in non-overlapping gray matter volumes (758functional subdivisions of90anatomical regions, averaging$1.7{\text{ cm}}^{3}$;Dornas & Braun, 2018). Other studies have used lower-dimensional*spatial*activity patterns in overlapping searchlight volumes ($O(10^{2})$voxels or dimensions, covering0.25to$1.0{\text{cm}}^{3}$;Kriegeskorte et al., 2006). Secondly, we employed multi-class linear discriminant analysis (“direct linear discriminant analysis,” DLDA;Yu & Yang, 2001), rather than pairwise discriminability or one-versus-all discriminability (e.g.,Hung et al., 2005;Liu et al., 2009). With these modifications, RSA revealed representational geometry at the level of object exemplars, as well as gradual changes in this geometry over sessions and runs.

#### 2.5.1 Linear discriminant analysis

To analyze the response variance that discriminatesκ=15recurring objects, at most(κ−1)-dimensions are required. Restricting the analysis to14principal components of the response could potentially have neglected smaller but more discriminating components. Accordingly, we performed a Linear Discriminant Analysis (LDA), which amounts to a “supervised” principal component analysis (PCA) and yields the(κ−1)-dimensional orthonormal subspaceSthat optimally discriminates theκresponse classes. Here, optimality is defined as simultaneously minimizing within-class variance and maximizing between-class variance of responses.

The results of LDA and PCA showed considerable commonality. Over the 758 parcels, the first 14 principal components captured53±7%(mean and S.D.) of the total response variance, whereas the 14-dimensional subspacesScaptured33±7%of the total variance (or61±6%of the principal component variance). Almost all of the subspace variance overlapped with the principal component variance (i.e.,88±5%of subspace variance projected into the space of the first 14 principal components, while the remaining12±5%projected into the space of the remaining principal components).

Similar numbers were obtained for the 124 identity-selective parcels. The first 14 principal components captured57±6%(mean and S.D.) of the total response variance, and subspacesScaptured38±6%of the total variance (or67±4%of the principal component variance). Almost all of the subspace variance (91±3%) overlapped with the first 14 principal components. In summary, Linear Discriminant Analysis captured the useful (discriminating) part of correlated variance and distributed this variance more uniformly over its 14 orthonormal dimensions (6±3%per dimension) than principal component analysis could (4±6%per dimension).

A numerically tractable procedure for identifying the optimal subspaceSis available in terms of “direct LDA” or DLDA (Ye et al., 2006;Yu & Yang, 2001). Briefly, this method first diagonalizes between-class variance to identifyκ−1discriminative eigenvectors with non-zero eigenvalues, next diagonalizes within-class variance, and finally yields a rectangular matrix for projecting activity patterns from the original activity space (dimensionality$N_{dim}$) to the maximally discriminative subspaceSand back. As this method is linear and relies on all available degrees of freedom, its results are deterministic. An important feature of this particular algorithm is that within-class variance is maintained near unity for all classes, by means of a suitable scaling of the subspace dimensions. The linkgithub.com/cognitive-biology/DLDAprovides a Matlab implementation of DLDA.

#### 2.5.2 Amplitudes, distances, and correlations

Activity patterns$x_{jk}$associated with trialskwere analyzed in the maximally discriminative subspaceS. The normalized amplitude$a_{k}=\sqrt{\frac{1}{\kappa -1}\sum_{j=1}^{\kappa -1}x_{jk}^{2}}$of such patterns exhibited an average value of〈a〉=0.99. The normalized distance$d_{kl}=\sqrt{\frac{1}{\kappa -1}\sum_{j=1}^{\kappa -1}{\left(x_{jk}-x_{jl}\right)}^{2}}$between patterns associated with trialskandlmeasured on average〈d〉=1.40, consistent with distance expected between random patterns of this amplitude ($\sqrt{2}$). Averaging over trialskproduced normalized response amplitudes$A={\langle a_{k}\rangle }_{k}$. Averaging over pairs of trialsk,lseparated by a given latencyl−k, produced normalized response distances$D(l-k)={\langle d_{kl}\rangle }_{k,l}$.

The patterns from successive trials exhibited a weak temporal correlation, with approximately5%smaller distances at delays below4trials and approximately2%larger distances at delays ranging from6to15trials (see SupplementaryFig. S1A, B). Comparing pairs of trials with different types of objects, we observed approximately3%*larger*response distancesD(at all latencies) for the same recurring objects than for either different recurring or non-recurring objects (SupplementaryFig. S1C). Differential response amplitudesAincreased marginally with latency, because response amplitudes tended to increase slightly over the course of each run (SupplementaryFig. S1D). This trend was evident for all types of objects and with both “structured” and “unstructured” sequences. In other words, the effect of object type on multivariate hemodynamic responses was limited to response distances and did not extend to response amplitudes. Thus, our data provided no evidence for “repetition suppression.”

For certain analyses (Sections 2.5.8and2.5.9), we established for each parcelwthe average delay-dependent distance$T_{w}(\Delta k)={\langle d_{w,u,r}(\Delta k)\rangle }_{u,r}$between patterns with a relative delay ofΔktrials, where the average was taken over subjectsuand runsr. The time-course$T_{w}$allowed us to discount temporal correlations by computing$d_{w,u,r}^{corrected}(\Delta k)=d_{w,u,r}(\Delta k)-T_{w}(\Delta k)+{\langle T_{w}(\Delta k)\rangle }_{\Delta k}$, where${\langle T_{w}(\Delta k)\rangle }_{\Delta k}$is the average value over delaysΔk.

#### 2.5.3 Representation of shape “identity” for recurring objects

Our observations comprised approximately200activity patterns for each of the15recurring object classes (per observer and condition). To allow for cross-validation, we randomly divided these patterns in a larger “training set” (90%or190±7.7per object class) and a smaller test set (10%or22±0.9per object class) (Fig. 2B). Note that the “training set” comprised exclusively activity patterns associated with recurring objects. To reduce the variability introduced by random test sets, this selection was repeated$N_{r}=20$times and all statistical measures described below represent the average over repetitions. As illustrated inFigure 2C, in the discriminative subspaceS, we compared the$n_{i}$test set exemplars$x_{ki}$(where$k=1,\ldots ,n_{i}$) of classito the centroids$c_{j}^{train}$established for the training exemplars of classj. To compute Mahalanobis distances and variance ratios (see below), we compared test set exemplars$x_{ki}$of classito the centroids$c_{j}^{test}$of test set exemplars of classj.

We used three measures for this comparison, all with comparable results. Firstly, the nearest class centroid$c_{i}^{train}$to each pattern exemplar$x_{ki}$was identified to establish a matrix of classification probabilitiesP(j​|i)(probability that an exemplar of classiis nearest to the centroid of classj), also known as “confusion matrix,” as well as the “classification accuracy”$\alpha =\sum_{i}P(i|i)P(i)$, which is the probability that the nearest centroid is the correct one.

Secondly, for each pair of object classes(i,j), object exemplars$x_{ki}$and$x_{kj}$from the test set were projected onto the line connecting the two test set centroids,$c_{i}^{test}$and$c_{j}^{test}$, and a pairwise discriminability/dissimilarity/Mahalanobis distance$\delta_{i,j}$was computed from the means,$\mu_{i}$and$\mu_{j}$, and variances,$\sigma_{i}^{2}$and$\sigma_{j}^{2}$, of these projections, as$\delta_{i,j}=\frac{\left|\mu_{i}-\mu_{j}\right|}{\sqrt{\frac{1}{2}\left(\sigma_{i}^{2}+\sigma_{j}^{2}\right)}}$. The average over all pairs of object classes was computed as$\delta =\frac{2}{\kappa \left(\kappa -1\right)}\sum_{i,j}\delta_{i,j}$.

Thirdly, given class centroids$c_{i}^{test}$and overall centroid$c^{test}$, we computed the Euclidean distances$d_{ki}=\left\|x_{ki}-c_{i}^{test}\right\|$between exemplars$x_{ki}$and class centroid$c_{i}^{test}$and, for each object classi, the “sum of squares” as$SS_{Wi}=\sum_{k=1}^{n_{i}}d_{ki}^{2}$. The “within-class” variance of all classes was computed as$SS_{W}=\frac{1}{N}\sum_{i=1}^{\kappa }SS_{Wi}$, where$N=\sum_{i=1}^{\kappa }n_{i}$. Similarly, from the Euclidean distances$d_{i}\;=\left\|c_{i}^{test}-c^{test}\right\|$between individual and overall centroids, we computed “between-class” variance$SS_{B}\;=\frac{1}{N}\sum_{i=1}^{\kappa }n_{i}d_{i}^{2}$. From the Euclidean distances$d_{ki}\;=\left\|x_{ki}^{test}-c^{test}\right\|$between exemplars and overall centroid, we computed “total” variance$SS_{T}\;=\frac{1}{N}\sum_{i=1}^{\kappa }\sum_{k=1}^{n_{i}}d_{ki}^{2}$. Variances$SS_{W}$,$SS_{B}$, and$SS_{T}$are also denoted, respectively,$SS_{same}$,$SS_{diff}$, and$SS_{fam}$further below. To quantify the discriminability of classes, the variance ratio$F_{identity}\;=SS_{B}(N-\kappa )/SS_{W}(\kappa -1)$provided a non-parametric multivariate statistic (PERMANOVA;Anderson, 2001). The average within-class and between-class dispersion per dimension could be estimated as$\sigma_{W}\;=\sqrt{SS_{W}/(N-\kappa )}$and$\sigma_{B}=\sqrt{SS_{B}/(\kappa -1)}$, respectively.

#### 2.5.4 Minimum statistic

To test for statistical significance, we computed average classification performance (in terms of both classification accuracy$\alpha_{obs}$and f-ratio$F_{obs}$) over$N_{r}$test sets, as well as over$10^{3}$first-level permutations of object identities (in each of the$N_{r}$test sets). In principle, we could have tested an “individual null” hypothesis for every parcel and every data set, namely, the probability of obtaining the observed performance$\alpha_{obs}$(or$F_{obs}$) purely by chance. Instead, we computed the “minimum statistic”$m=min_{k}\alpha_{k}$(or$m=min_{k}F_{k}$) over data setsk, as well as over$10^{5}$second-level permutations (drawn randomly from the first level permutations) and tested the “global null” hypothesis, namely, the probability$p_{n}(m)$of obtaining the observed minimum performance overndata sets purely by chance. This computation was performed separately for each of the 2 conditions (8 data sets from 8 observers per condition) as well as for the union of conditions (16 data sets from 8 observers). When the “global null” hypothesis could be rejected, we inferred statistically significant classification performance in at least*some*data sets. Our threshold for significance was$p_{n}^{⋆}(m)<0.05$after correction for multiple comparisons (758parcels and2conditions) (Allefeld et al., 2016).

#### 2.5.5 Prevalence analysis

To summarize the results from all observers and conditions, we used a “prevalence analysis” (Allefeld et al., 2016). Prevalence$\gamma_{true}$is the fraction of significant performance overn=16data sets. To test the “prevalence null” hypothesis that$\gamma_{true}$is below a threshold$\gamma_{0}\;=0.5$, an upper bound for$P(\gamma_{true}<\gamma_{0})$was obtained from the probability$p_{n}^{⋆}(m)$of the minimum statistic overn=16data sets, after correction for multiple comparisons:

$$P(\gamma_{true}\;<\gamma_{0})\leq p_{n}(m|\gamma )={\left[(1-\gamma_{0})\;\sqrt[{n}]{p_{n}^{⋆}\left(m\right)}+\gamma_{0}\right]}^{n}$$

This was the criterion used to label parcels as “identity selective.” Threshold prevalenceγ≃0.5corresponded to corrected probability$p_{n}^{⋆}(m)≃0.0012$and*minimal*accuracy of6.67%(i.e., near chance).

Additionally, we computed$\gamma_{est}$as the largest value for which the “prevalence null” hypothesis could be rejected from

$$\gamma_{est}=\frac{\sqrt[{n}]{\alpha }-\sqrt[{n}]{p_{n}^{⋆}(m)}}{1-\sqrt[{n}]{p_{n}^{⋆}(m)}}$$

where$p_{n}^{⋆}(m)$is the corrected minimum probability,n=16the number of data sets, andα=0.05the significance threshold.

#### 2.5.6 Representation of shape “novelty” for non-recurring objects

Although recurring and non-recurring objects were comparable and generated in the same way, it seemed possible that neural representations might discriminate the class of 15 recurring objects from the class of 360 non-recurring objects. Indeed, the two classes became discriminable after observers had learned to classify recurring objects as “familiar” and non-recurring objects as “novel.” Accordingly, we considered this discriminability a representation of “novelty.”

To assess the neural representation of “novelty,” we divided non-recurring and recurring objects into two sets of unequal size (approximatelyN=216×15recurrent or “familiar” exemplars vs.M=360non-recurrent or “novel” exemplars). From the Euclidean distances$d_{k}\;=\left\|x_{k}\;-c\right\|$between test set exemplars$x_{k}$and centroids$c_{fam}\;=\frac{1}{N}\sum_{k=1}^{N}x_{k}$or$c_{nov}\;=\frac{1}{M}\sum_{k=1}^{M}x_{k}$, we obtained “within-class” variance$SS_{W}\;=SS_{fam}\;+SS_{nov}$, where$SS_{fam}\;=\frac{1}{N+M}\sum_{k=1}^{N}d_{k,fam}^{2}$and$SS_{nov}\;=\frac{1}{N+M}\sum_{k=1}^{M}d_{k,nov}^{2}$. From distances$d_{fam}\;=\left\|c_{fam}\;-c_{tot}\right\|$and$d_{nov}\;=\left\|c_{nov}\;-c_{tot}\right\|$between class centroids and overall centroid$c_{tot}\;=\frac{N}{N+M}c_{fam}\;+\frac{M}{N+M}c_{nov}$, we obtained “between-class” variance$SS_{B}\;=SS_{novfam}=\frac{N}{N+M}d_{fam}^{2}+\frac{M}{N+M}d_{nov}^{2}=$$\frac{NM}{{\left(N+M\right)}^{2}}{\left(c_{fam}\;-c_{nov}\right)}^{2}$. Finally, from distances$d_{k}\;=$$\;\left\|x_{k}\;-c_{tot}\right\|$between exemplars and overall centroid, we obtained total variance$SS_{T}=\frac{1}{N+M}\sum_{k=1}^{N+M}d_{k}^{2}$. To quantify the discriminability of non-recurring and recurring objects, we formed the variance ratio$F_{novelty}\;=SS_{B}(N+M\;-2)/SS_{W}$(Anderson, 2001). Average within-class and between-class dispersion per dimension was obtained from$\sigma_{W}\;=\sqrt{SS_{W}/(N+M-2)}$and$\sigma_{B}=\sqrt{SS_{B}}$, respectively.

#### 2.5.7 Changes of representation analyzed in “batches”

To assess changes in neural representations over the course of the experiment, while also allowing for cross-validation, we divided all recurring object presentations into five successive “batches”$B_{1},B_{2},\ldots$, each with20%of the presentations (Fig. 2D). In this way, we could select “test sets” for cross-validated DLDA from one particular batch, while retaining all other presentations as a “training set.” As every recurrent object was presented210±9times over all sessions, a batch would comprise42±1.8presentations, a test set21±0.9, and a training set189±8.1presentations. To reduce the variance deriving from test set selection, we repeated the random selection$N_{r}\;=20$times and averaged over repetitions.

To quantify representational changes over the course of learning, we computed the variance ratios$F_{m,w,u}^{identity}$for each temporal window or batchm, identity-selective parcelw, and data setsu∈{1,…,16}. We formed the average ratio over 16 data sets,$F_{m,w}^{identity}\;={\langle F_{m,w,u}^{identity}\rangle }_{u}$, and assessed statistical significance by shuffling ($10^{3}$permutations) the identity of recurring objects to obtain the distribution of variance ratios due to chance or data structure. The mean$\mu_{m,w}$and variance$\sigma_{m,w}^{2}$of this distribution could also be used to convert$F_{m,w}^{identity}$into z-score values$Z_{m,w}^{identity}=\left(F_{m,w}^{identity}\;-\mu_{m,w}\right)/\sigma_{m,w}$.

Additionally, we performed a regression analysis and quantified representational changes in terms of linear trends. Specifically, we determined a “rate” parameter$\beta_{w}^{identity}$by fitting a linear mixed-model$F_{m,w,u}^{identity}\;=$$\beta_{0,w}\;+\beta_{w}^{identity}m+\xi_{0,w,u}\;+\xi_{1,w,u}m+\epsilon_{m,w,u}$with data setsuas the grouping variable, where$\beta_{0,w}$was a fixed-effect coefficient,$\xi_{0,w,u}$and$\xi_{1,w,u}$were random effect coefficients, and$\epsilon_{m,w,u}$was residual error.

Similarly, to assess whether neural representations of non-recurring objects change with learning, we divided all object presentations (recurring and non-recurring) into five successive “batches”$B_{1},B_{2},...$, each with20%of the presentations (Fig. 2D), to obtain variance ratios$F_{m,w,u}^{novelty}$for each temporal window or batchm, identity-selective parcelw, and data setsu∈{1,…,16}. After averaging over 16 data sets,$F_{m,w}^{novelty}={\langle F_{m,w,u}^{novelty}\rangle }_{u}$, we assessed statistical significance by shuffling ($10^{3}$permutations) the identity of recurring and non-recurring objects to obtain the distribution of variance ratios due to chance or data structure. The mean$\mu_{m,w}$and variance$\sigma_{m,w}^{2}$of this distribution were used to convert$F_{m,w}^{novelty}$into z-score values$Z_{m,w}^{novelty}\;=\left(F_{m,w}^{novelty}\;-\mu_{m,w}\right)/\sigma_{m,w}$.

Additionally, we performed a regression analysis to establish linear trends. Changes in the representation of object “novelty” were assessed by fitting the “rate” parameter$\beta_{w}^{novelty}$in a linear mixed-model$F_{m,w,u}^{novelty}\;=\beta_{0,w}\;+\beta_{w}^{novelty}m+\xi_{0,w,u}\;+\xi_{1,w,u}m+\epsilon_{m,w,u}$, with data setsuas the grouping variable, where$\beta_{0,w}$was a fixed-effect coefficient,$\xi_{0,w,u}$and$\xi_{1,w,u}$were random effect coefficients, and$\epsilon_{m,w,u}$was a residual error.

To establish linear trends$F_{m}\;={\langle F_{m,w,u}\rangle }_{w,u}$(of either identity and novelty) that average over both parcelswand data setsu, we obtained a rate parameter$\beta_{1}$by fitting linear mixed-model$F_{m,w,u}\;=\beta_{0}\;+\beta_{1}m+\xi_{0,w,u}\;+\xi_{1,w,u}m+\epsilon_{m,w,u}$with both parcels and data sets as grouping variables.

#### 2.5.8 Geometry of representations

In the cross-validated analyses described above, subspacesSdiffered slightly between different batches (and training sets). To analyze the geometry of neural representations in a stable framework, we repeated some analyses in fixed subspacesSthat reflected all observations (i.e., all recurring activity patterns$x_{k}$). In the fixed subspace, we calculated the normalized amplitude$a_{k}\;=\left\|x_{k}\right\|/\sqrt{\kappa -1}=\sqrt{\sum_{j=1}^{\kappa -1}x_{jk}^{2}}/\sqrt{\kappa -1}$of individual patternskand the normalized pairwise distance$d_{kl}\;=\left\|x_{k}-x_{l}\right\|/\sqrt{\kappa -1}=\sqrt{\sum_{j=1}^{\kappa -1}{\left(x_{jk}-x_{jl}\right)}^{2}}/\sqrt{\kappa -1}$between two patternskandl.

For each parcelw, data setu, and runr, we obtained the average amplitude$A_{w,u,r}^{tot}\;=\frac{1}{N+M}\sum_{k=1}^{N+M}a_{k}$of all patterns, the average amplitude$A_{w,u,r}^{fam}\;=\frac{1}{N}\sum_{k=1}^{N}a_{k}$of*recurring*patterns, and the average amplitude$A_{w,u,r}^{nov}\;=\frac{1}{M}\sum_{k=1}^{M}a_{k}$of*non-recurring*patterns. Similarly, we obtained the average pairwise distance$D_{w,u,r}^{tot}\;=\frac{2}{\left(N+M)(N+M-1\right)}$$\sum_{k=1}^{N+M}\sum_{l=k}^{N+M}d_{kl}$between all patterns, the average distance$D_{w,u,r}^{nov}=\frac{2}{M(M-1)}\sum_{k=1}^{M}\sum_{l=k}^{M}d_{kl}$between non-recurring patterns, the average distance$D_{w,u,r}^{fam}\;=$$\frac{2}{N(N-1)}\sum_{k=1}^{N}\sum_{l=k}^{N}d_{kl}$between recurring patterns, and the average distance$D_{w,u,r}^{novfam}\;=\frac{1}{MN}\sum_{k=1}^{M}\sum_{l=1}^{N}d_{kl}$between pairs comprising one recurring and one non-recurring pattern. For recurring patterns, we further obtained the average distance$D_{w,u,r}^{same}=\frac{2}{N(N/\kappa -1)}\sum_{i=1}^{\kappa }\sum_{k=1}^{n_{i}}\sum_{l=k}^{n_{i}}d_{kl}$between pairs of recurring patterns in the*same*class and the average distance$D_{w,u,r}^{diff}\;=\frac{1}{N(N-N/\kappa )}\sum_{i=1}^{\kappa }\sum_{k=1}^{n_{i}}$$\sum_{l=1}^{N-n_{i}}d_{kl}$between pairs in*different*classes. All distances were corrected for the temporal auto-correlation by subtracting the time course of$T_{w}(i,j)$, as described above.

As described further above, the distances between individual activity patterns and different centroids—such as$c_{tot}$,$c_{nov}$, and$c_{fam}$—yielded total variance$SS_{T}\;=SS_{tot}$, within-class variance$SS_{W}\;=SS_{fam}\;+SS_{nov}$, and between-class variance$SS_{B}\;=SS_{novfam}$. For recurring patterns, distances to individual class centroids$c_{i}$and overall centroid$c_{fam}$yielded total variance$SS_{T}\;=SS_{fam}$, within-class variance$SS_{W}\;=SS_{same}$, and between-class variance$SS_{B}=SS_{diff}$.

These values were computed for each parcelw, observeru, and runr, in order to obtain variance fractions$F_{w,u,r}^{fam}\;=SS_{fam}/SS_{tot}$,$F_{w,u,r}^{nov}\;=SS_{nov}/SS_{tot}$,$F_{w,u,r}^{novfam}\;=$$\;SS_{novfam}/SS_{tot}$,$F_{w,u,r}^{same}\;=SS_{same}/SS_{fam}$, and$F_{w,u,r}^{diff}=SS_{diff}/$$SS_{fam}$, as well as variance ratios$R_{w,u,r}^{identity}\;=SS_{diff}(N-\kappa )/$$SS_{same}(\kappa -1)$and$R_{w,u,r}^{novelty}\;=SS_{novfam}(N+M-2)/\;(SS_{nov}\;\;=$$SS_{fam})$.

#### 2.5.9 Changes with learning analyzed by “runs”

Fixed subspaces permitted us to assess representational changes between successive “runs.” To this end, we computed average amplitudes$A_{w,u,r}$, distances$D_{w,u,r}$, variances$SS_{w,u,r}$, and variance ratios$F_{w,u,r}$, as described above, for each parcelw, data setu∈{1,…,16}, and runr. Within each sessions, we assessed the changes of these parametersY ​∈{A,D,SS,F}over runs$r^{\prime }\in s$by determining a “rate” parameter$\beta_{s}$for identity-selectivewand non-selective parcels$w^{\prime }$. Each$\beta_{s}$coefficient was acquired from a linear mixed-model$Y_{r^{\prime },w,u}\;=\beta_{0,s}\;+\beta_{s}r\prime +\xi_{0,w,u}\;+\xi_{1,w,u}r\prime \;+\epsilon_{r^{\prime },w,u}$with observers and parcels as grouping variables, where$\beta_{0,s}$was a fixed-effect coefficient,$\xi_{0,w,u}$and$\xi_{1,w,u}$were random effect coefficients, andϵwas residual error. The same approach was used to assess gradual changes over runs in the centroid-to-centroid distances$D_{same}(r)$,$\Delta D_{same}(r)$,$D_{nov}(r)$, and$\Delta D_{nov}(r)$. This served to test the statistical significance of linear rates$\beta_{s}$in each session. Sessions with significant rates are marked by stars inFigure 6.

#### 2.5.10 Stability of shape identity and novelty representations

We also assessed the stability of the representation of the 16 response classes (15 recurring and 1 non-recurring) over the course of the experiment. To this end, we compared the average representation in individual runsr(centroids$C_{r}$of responses to exemplars) to the average representation over all runs (centroids$C_{ave}$). For observeru, identity-selective parcelw, and object classi, we calculated the Euclidean distance$D_{u,w,i,r}$between the relevant$C_{r}$and$C_{ave}$, and also the difference$\Delta D_{u,w,i,r}$between the relevant centroids from successive runs,$C_{r}$and$C_{r+1}$. After averaging over observersu, identity-selective parcelsw, and object classesi, we obtained$D_{same}(r)$and$\Delta D_{same}$for recurring objects and by$D_{nov}(r)$and$\Delta D_{nov}(r)$for non-recurring objects.

As a baseline for comparison, we also computed the distances$D_{u,w,i,r}$and differences$\Delta D_{u,w,i,r}$that may be expected purely on the basis of response variance. To this end, we permuted the sequence of all3,600trials, separately within each of the 16 response classes (15 recurring and 1 non-recurring) such as to obtain 18 “pseudo-runs” with200trials each. Expectation values were obtained by repeating this$N_{r}\;=1,000$times.

We note that, in ann-dimensional hypersphere of unit radius, the average Euclidean distance between two random points is

$$d_{ave}=\frac{2^{n}}{\sqrt{\pi }}\frac{\Gamma^{2}\left(\frac{n+1}{2}\right)}{\Gamma \left(n+\frac{1}{2}\right)}$$

with$d_{ave}\approx 1.4017$forn=14.

#### 2.5.11 Dimensional reduction

To visualize representational geometry in two dimensions, we randomly sampled50response patterns to each of the recurring and non-recurring objects within the first and the last sessions and calculated a1,600×1,600pair-wise distance matrix ($D_{w,u}$) for each identity-selective parcelwand subjectu. We did not wish to average distance matrices over observers, as we did not expect the activity patterns of different observers to be comparable. To sidestep this difficulty, we permuted the order of recurring objects100times and for each subject obtained an average matrix$\bar{D}$over permutations, which was then averaged over subjects. To visualize the representational geometry of identity in the first and the last session, we used multidimensional scaling (Matlab function*mdscale*, metric stress) to map the distances matrices for recurring objects (50exemplars from the first session and50exemplars from the last session) into a two-dimensional space. To visualize the representational geometry of novelty, we restricted the distance matrix to non-recurring objects (50exemplars from the first session and50exemplars from the last session) and just 3 of the 15 recurring objects (20exemplars from either session).

## 3 Results

Observers viewed sequences of computer-generated objects, with each object shown for2.5swhile rotating in three dimensions (Fig. 1A, B, a movie may be viewedHERE). Over three sessions, observers viewed 3,600 objects in total, of which 3,240 were presentations of*recurring*objects (15 different objects, each appearing approximately 216 times) and 360 were presentations of*non-recurring*objects (360 different objects, each appearing once). The display was intended to be sufficiently intriguing to remain interesting over 3 successive days. To this end, presentations never repeated exactly. Observers were required to classify each object as “familiar” (recurring) or “novel” (non-recurring). The task performance improved as observers became increasingly familiar with recurring objects, as illustrated inFigure 1C. Over the first 600 presentations, classification performance improved approximately from50%correct (chance) to85%correct, and reaction times decreased approximately from1.65 sto1.25 s. Over the remaining 3,000 presentations, performance improved further to approximately90%correct and reaction times decreased further to approximately0.95 s. After three sessions, all observers were “familiar” with all recurring objects and could pick them out from an array of distractor objects.

All sessions were performed in an MRI scanner while whole-brain functional imaging data were being collected. In the following, we report the results of three types of analyses. First, we describe the cortical areas in which multivariate BOLD activity encodes information about the identity of recurring objects (“object identity”), as determined by cross-validated analyses of entire data sets (3 sessions per observer). Second, we describe changes in cortical representations over coarse time intervals, by means of cross-validated analyses of successive parts of the data sets (3 sessions divided into 5 batches). These changes pertain to the encoding of both recurring objects and the distinction between recurring and non-recurring objects (“object novelty”). Third, we describe changes in representations over finer time intervals (3 sessions divided into 18 runs), by foregoing cross-validation and adopting a fixed reference frame. These finer intervals confirm the results from coarse intervals but reveal more details about the geometry of neural representations and their development over time.

### 3.1 Cross-validated representation of object identity

**Fig. 3.**
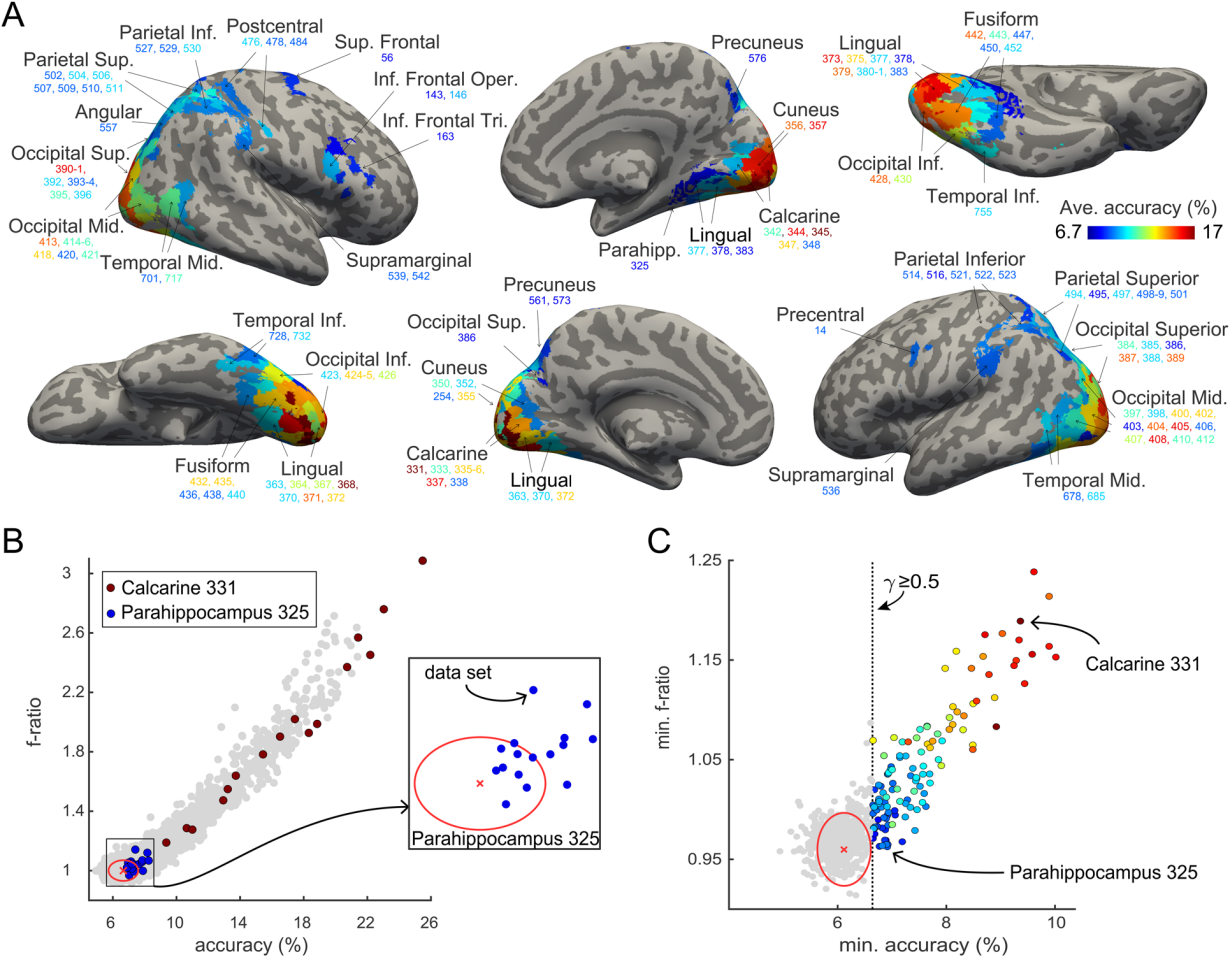
Neural representation of object identity. (A) Identity-selective parcels are shown in color (124 of 758 parcels) on an inflated standard brain and are found in the occipital (70 parcels), parietal (29), temporal/fusiform (18), and frontal cortex (7). Color indicates classification$\alpha_{w}^{ave}$(average over 16 data sets), and ranges from chance to the largest observed value (6.67%to17%). Parcels are identified by AAL region and number (in color), as detailed inAppendix Table A1. (B) Classification$\alpha_{w,u}$and variance ratio$F_{w,u}$for all 758 parcelswand 16 data setsu. Both values differ highly significantly from the values obtained with shuffled object identities (red cross and ellipse, representing mean±3 S.D.). Two particular parcels are highlighted (Calcarine-L 331 in red, Parahippocampus-R 325 in blue, and magnified in the inset) to illustrate the variability of data sets. (C) Minimum values$a_{w}^{min}$and$F_{w}^{min}$for all parcels over 16 data sets. Identity-selective parcels are colored according to$a_{w}^{ave}$as in (A). A minimum above chance6.67%corresponds to a prevalenceγabove0.5(dotted vertical line). The distributions obtained with shuffled identities are indicated as well (red cross and ellipse).

To assess the extent to which multivariate neural responses to recurring objects encoded object identity, we relied on optimal linear classifiers combined with cross-validation (“direct linear discriminant analysis,” DLDA, seeMethodsfor details). Specifically, we quantified the “identity” information in multivariate responses of every parcelw∈{1,…,758}and data setu∈{1,…,16}in terms of classification accuracy$\alpha_{w,u}$, average pairwise dissimilarity$\delta_{w,u}$, and the ratio of between-class and within-class variance$F_{w,u}$. All three measures proved highly correlated and supported similar conclusions. For example,Figure 3Billustrates the correlation of classification accuracy$\alpha_{w,u}$and variance ratio$F_{w,u}$(ρ=0.94,p<0.001). The correlations of$a_{w,u}$and$\delta_{w,u}$(ρ=0.95,p<0.001), and of$\delta_{w,u}$and$F_{w,u}$(ρ=0.98,p<0.001) were comparably strong. The results of individual observers from the two experimental conditions (structured and unstructured object sequences) were highly similar as well, demonstrating test-retest consistency (SupplementaryFig. S2).

For most parcels, the results from different observers showed considerable variability. Whereas a few parcels exhibited significant accuracy$\alpha_{w,u}$and variance ratio$F_{w,u}$in all data sets (e.g., Calcarine 331), in many parcels the representation of object identity was significant only in some data sets (e.g., Parahippocampus 325) (Fig. 3B). Global significance was assessed by comparing the*minimal*accuracy or variance ratio over the 8 data sets from one condition (structured or unstructured) to the minimal values obtained with shuffled data (red ellipse inFig. 3C, seeMethodsfor details).

Minimal classification accuracy$\alpha_{w}$was significant in 17% of all parcels (128 of 758 parcels) in the structured sequence condition and in 19% of parcels (146 of 748) in the unstructured condition ($p^{⋆}\;0.05$, corrected for multiple comparisons), when compared to null-distributions obtained from shuffled object identities. For minimal variance ratios$F_{w,u}$, the corresponding values were 18% and 17%, respectively (136 and 130 parcels). To combine the results from both conditions, we used a “prevalence” analysis to determine parcels in which “identity” was represented significantly in a majority of all 16 data sets (prevalenceγ≥0.5), once again comparing the observed minimal values to the minimal values obtained with shuffled data (red ellipse inFig. 3C, seeMethodsfor details).

Figure 3Aillustrates the 124 parcels identified as significantly “identity-selective” by the prevalence criterionγ≥0.5and SupplementaryFigure S3shows the same information in terms of a sliced brain. Among these were70parcels in the occipital cortex,29in the parietal cortex,18in the fusiform or temporal cortex, and7in the frontal cortex. The average prevalence of identity-selectivity in these parcels was0.663±0.016(mean and S.D.), and the minimal value was0.58. As the prevalence criterion (based on 16 data sets) was marginally more conservative than the accuracy criterion (based on 8 data sets), 120 of the 124 parcels were significantly “identity-selective” in terms of both criteria. The four exceptions (identified only by prevalence, but not by accuracy) were Frontal-superior-R 56, Occipital-superior-R 393, Occipital-middle-L 403, and Parietal-superior-R 510.Appendix Table A1lists the statistical significance of all three criteria for all “identity-selective” parcels.

Overall, there was a pronounced posterior-anterior gradient. Whereas many parcels at the posterior pole of the brain exhibited high classification accuracy, this tended to progressively decrease at more anterior locations (Fig. 3A; SupplementaryFig. S3;Appendix Table A1). To formalize this trend, we assigned 66 of the 124 identity-selective parcels to the 25 topographic visual areas defined byWang et al. (2015)and, additionally, to the anterior inferior temporal cortex (AIT) and to the inferior frontal cortex (IFC). SupplementaryFigure S6provides an overview of all topographically assigned and non-assigned parcels selective for identity. As illustrated inFigure 8A, this assignment showed that accuracy was comparable in early visual areas (V1-hV4) and in the posterior-ventrolateral regions of the temporal lobe, whereas accuracy was lower in the anterior temporal cortex, the inferior frontal cortex, and in parietal cortical areas.

### 3.2 Cross-validated changes with learning

To assess changes with learning, we separately analyzed five successive and non-overlapping sets of trials (“batches”) with linear classifiers and cross-validation (seeMethodsfor details). Specifically, we established ratios of between- and within-class variance for both object identity (15 classes formed by responses to 15 recurring objects) and for object novelty (2 classes formed by responses to recurring and non-recurring objects, respectively). These two variance ratios measured the neural representation of “identity” and “novelty.”

**Fig. 4.**
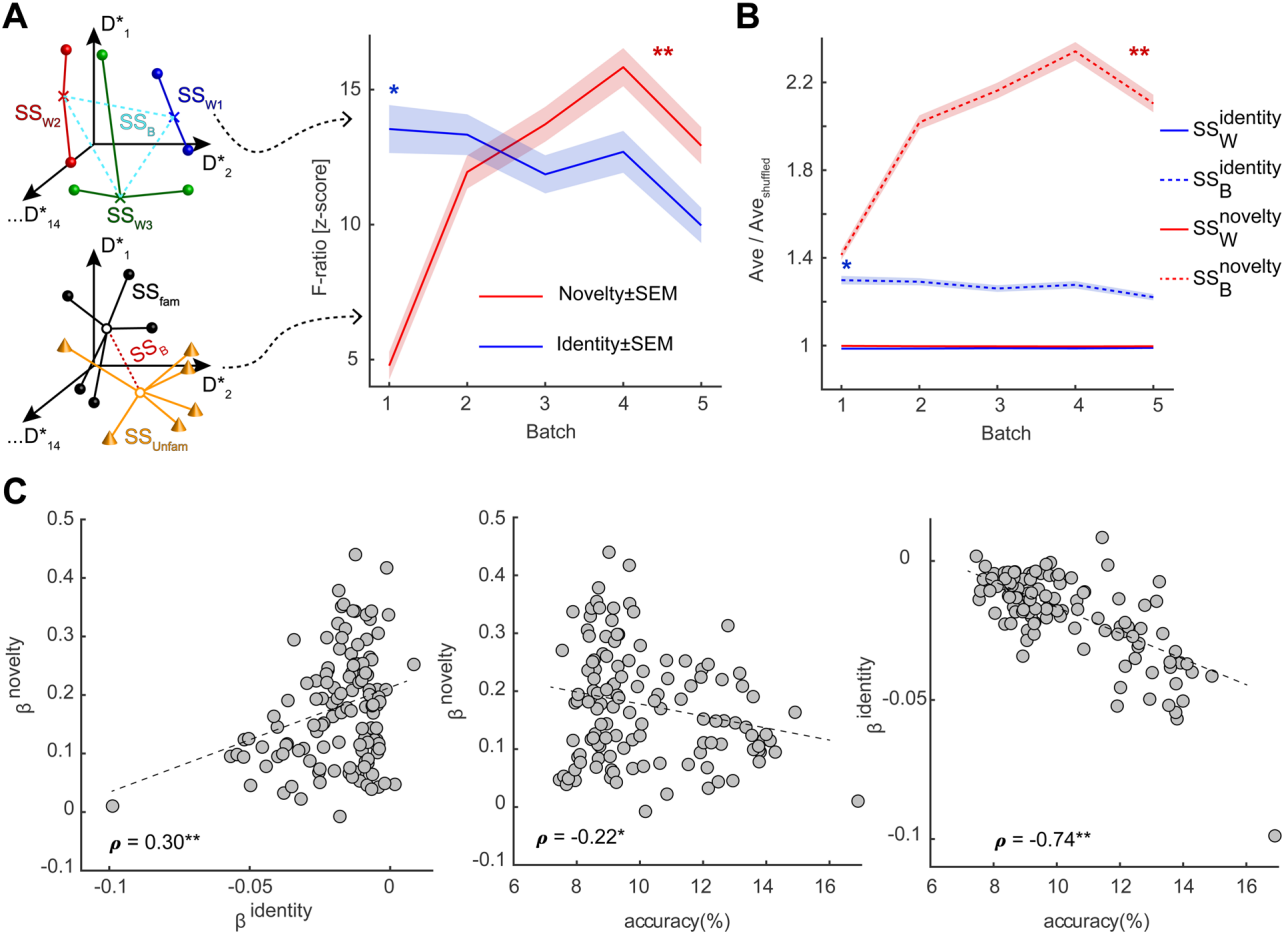
Changes in the representation of “identity” and “novelty” over successive “batches” of trials. (A) Ratio of within- and between-class variance for object “identity” (κ=15classes, inset top left) and object “novelty” (2 classes, inset bottom left). Average variance ratios$F_{m}^{identity}$(blue, mean±S.E.M.) and$F_{m}^{novelty}$(red, mean±S.E.M.), as a function of batch numberm. While$F_{m}^{identity}$decreases slightly over time (p<0.05),$F_{m}^{novelty}$increases considerably (p<0.001), especially initially. All values are averages over data sets in z-score units. (B) Average within- and between-class variances (mean±S.E.M.), as a function of batch numberm. Whereas between-class variances decrease ($SS_{B,m}^{identity}$,p<0.05) or increase ($SS_{B,m}^{novelty}$,p<0.001), within-class variances remain unchanged. All values are averages over data sets, relative to shuffled averages. (C) Results of regression analysis for 124 identity-selective parcelsw. Linear “rate” parameters$\beta_{w}^{identity}$and$\beta_{w}^{novelty}$compared to each other and to classification$\alpha_{w}$. Novelty and identity rates correlate weakly over parcels (left,ρ=0.298,p<0.001), as do novelty rate and classification accuracy$\alpha_{w}^{identity}$(middle,ρ=− 0.22,p<0.05). Identity rates$\beta_{w}$and accuracies$\alpha_{w}$correlate strongly and negatively (right,ρ=− 0.74,p<0.001). Significance of linear trends is indicated by * forp<0.05and ** forp<0.001.

Variance ratios were converted to z-score values (with respect to the mean and variance of the corresponding shuffle distribution) before being averaged over data sets and/or over parcels.Figure 4Asummarizes the results in terms of a grand average over all identity selective parcels. The average identity and novelty ratios were highly significant in all batches (p<0.001). Over successive batches, the average identity ratio weakened slightly but significantly (p<0.05), whereas the average novelty ratio strengthened considerably, especially between batchesm=1andm=2(p<0.001).

As expected, it was the between class-variances$SS_{B}^{identity}$and$SS_{B}^{novelty}$that changed significantly over successive batchesm(p<0.05andp<0.001, respectively), whereas the within-class variances$SS_{W}^{identity}$and$SS_{W}^{novelty}$remained essentially the same (p=n.s.), as illustrated byFigure 4B. This was owing to the DLDA algorithm, which maintained within-class variance near unity. Nevertheless, over successive batches, the neural representations of recurring objects tended to become slightly more similar to each other, but more dissimilar to the representations of non-recurring objects.

To ascertain that these overall trends hold true also for individual parcels, we carried out more conventional regression analyses of variance ratios$F_{m,w,u}^{identity}$and$F_{m,w,u}^{novelty}$over batchesm, parcelswand data setsu. Specifically, we fitted linear mixed-models in order to estimate “rate” parameters$\beta_{w}^{identity}$and$\beta_{w}^{novelty}$for each identity-selective parcelw. The results revealed negative rates$\beta_{w}^{identity}$and positive rates$\beta_{w}^{identity}$for almost all parcels, confirming the overall trends inFigure 4C. The variability over parcels was numerically larger for$\beta_{w}^{novelty}$(0.15±0.1, mean and S.D.) than for$\beta_{w}^{identity}$(0.022±0.015), with both rates weakly correlated (ρ=0.30,p<0.001). Classification accuracy$\alpha_{w}^{identity}$correlated negatively with$\beta_{w}^{novelty}$(ρ=− 0.22,p<0.05) and with$\beta_{w}^{identity}$(ρ=−​ 0.74,p<0.001).

**Fig. 5.**
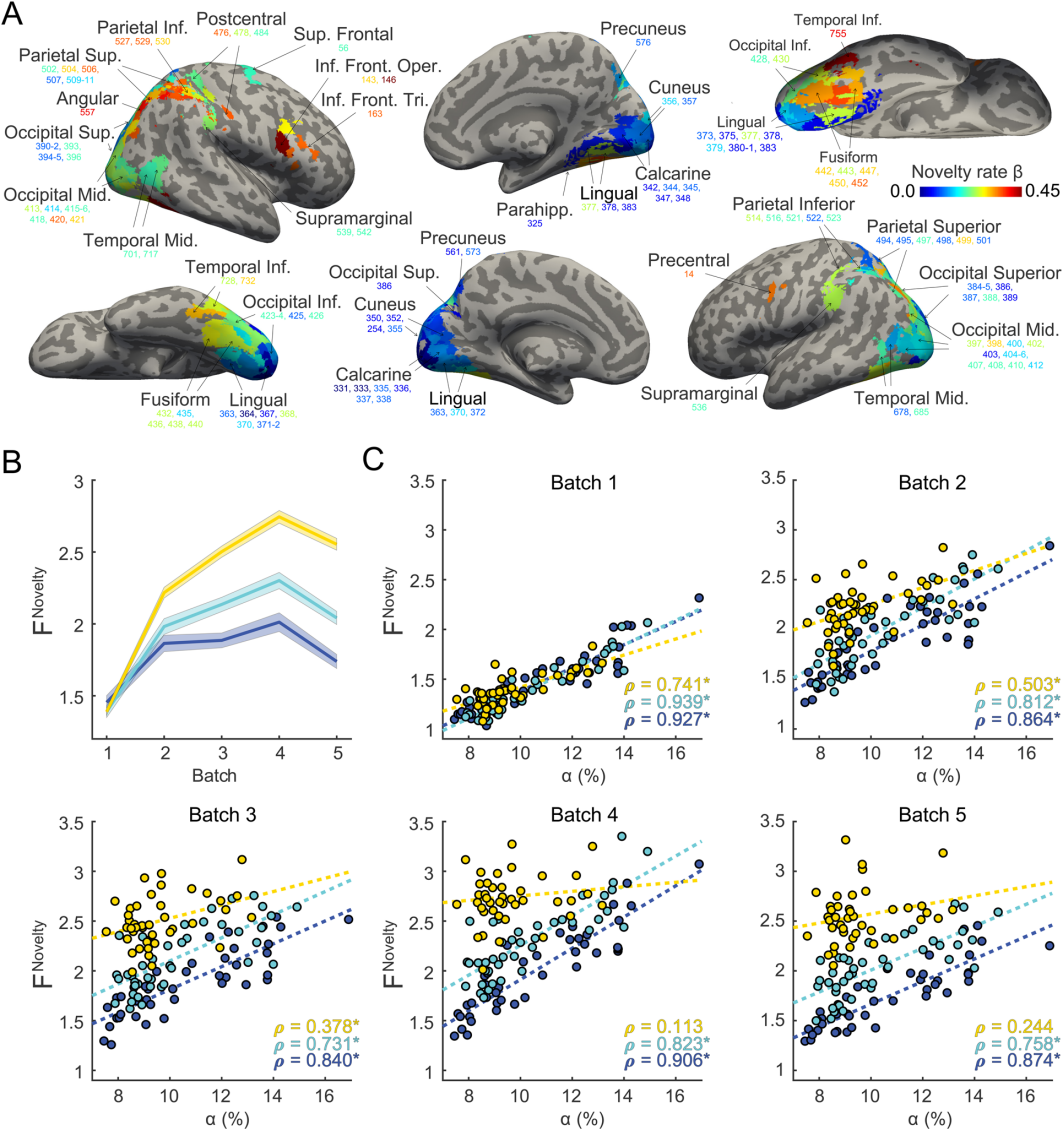
Neural representation of “novelty” in terms of the variance ratio$F^{novelty}$and its development over successive batches. (A) Identity-selective parcels and their individual rate parameters$\beta^{novelty}$(color scale), estimated by fitting linear-mixed models to the$F^{novelty}$values (from all batches and data sets). (B) Development of$F^{novelty}$(mean±S.E.M.) for different “novelty terciles” (upper, middle, and lower tercile of parcels defined by$\beta^{novelty}$). (C) Correlation between$F_{w}^{novelty}$and accuracy$\alpha_{w}$for different batches and novelty terciles. The parcels of each tercile are distinguished by color, with individual regression lines (dashed) and correlation coefficientsρ(⋆indicatesp<0.05).

To take a closer look at the interaction between “novelty” and “identity,” we divided the identity-selective parcels into “novelty terciles” (high, medium, and low, defined by$\beta^{novelty}$) before comparing representations of novelty ($F^{novelty}$) and identity (accuracyα) (Fig. 5B). The results differed substantially between batches and terciles. In early batches,$F^{novelty}$andαcorrelated for all terciles, suggesting that initially the representations of non-recurring and recurring objects were linked. However, in successively later batches, this correlation waned in the upper tercile. This may suggest that pronounced representations of non-recurrent objects progressively detached from representations of recurrent objects.

Figure 5Aillustrates the degree to which identity-selective parcels express the overall novelty trend, as quantified by fitted rate$\beta_{w}^{novelty}$, and SupplementaryFigure S4shows the same information in terms of brain slices. An anterior-posterior gradient is evident, with a more pronounced representation of novelty at anterior than at posterior locations. This gradient is also apparent when parcels are assigned to topographic visual areas, as illustrated inFigure 8B.Appendix Table A1lists the rates$\beta_{w}^{novelty}$for all identity-selective parcels.

### 3.3 Geometry of identity and novelty representations

Next, we present results from alternative analyses relying on fixed subspacesSfor each data set (3,600 trials). Fixed subspaces reveal a more detailed geometry of neural representations and allow any changes in this geometry to be tracked over successive runs (200 trials each). The disadvantage of this approach is that it precludes cross-validation. Our aim was to establish not just between- and within-class variances, but also the distances underlying the variances, and the response amplitudes underlying the distances. For the representation of object “identity,” the within- and between-class geometry was defined by response pairs to*same*and to*different*recurring objects, respectively. For the representation of object “novelty,” the within-class geometry reflected responses either to pairs of*familiar*(recurring) or to pairs of*novel*(non-recurring) objects, whereas the between-class geometry concerned responses to mixed pairs of objects (*novel-familiar*).

We analyzed multivariate responses in terms of variances, distances, and amplitudes and averaged the results over all data sets and all 124 identity-selective parcels, to obtain separate mean values (and standard errors) for each of the 18 successive runs. Additionally, we averaged the results over the remaining 634 (non-identity-selective) parcels of the brain. We hoped that this would help distinguish more general effects and trends (e.g., habituation, attention, alertness) from learning-related changes in shape representations. All distances in these analyses were residual distances, to minimize the influence of temporal auto-correlations (SupplementaryFig. S1; seeMethodsfor details).

**Fig. 6.**
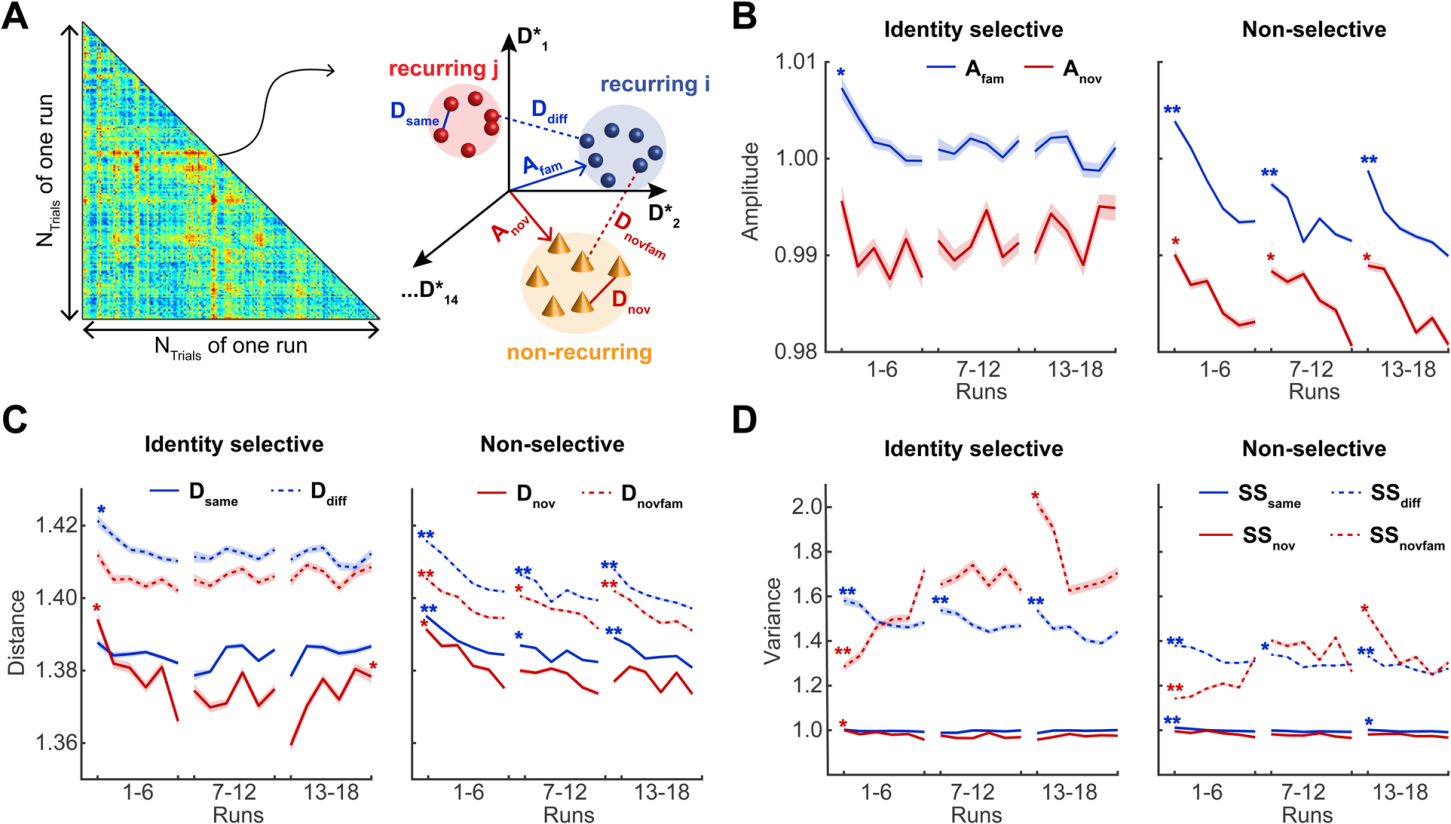
Geometry of identity and novelty representation over successive sessions and runs. (A) For each run with$N_{trials}=200$trials, we collected all individual response amplitudesaand all pairwise response distancesd(triangular area with color scale) in the maximally discriminating space and computed average amplitudes$A_{fam}$and$A_{nov}$(for recurring and non-recurring objects, respectively) and average distances$D_{same}$and$D_{diff}$(for same and different recurring objects, respectively), as well as average distances$D_{nov}$and$D_{novfam}$(for non-recurring objects and between recurring and non-recurring objects, respectively). (B) Response amplitude$A_{nov}$(red, mean, and S.E.M.) and$A_{fam}$(blue, mean, and S.E.M.), over 18 runs grouped into three sessions, for identity-selective (left) and non-selective parcels (right). (C) Pairwise response distance$D_{same}$(solid blue),$D_{diff}$(dashed blue),$D_{nov}$(solid red), and$D_{novfam}$(dashed red)), over runs and sessions, for both groups of parcels. (D) Variance of response distances$SS_{same}$(solid blue),$SS_{diff}$(dashed blue),$SS_{nov}$(solid red), and$SS_{novfam}$(dashed red), over runs and sessions, for identity-selective and non-selective parcels. Stars indicate a significant linear trend during a session (see text). All plots show mean (traces) and S.E.M. (shading).

The analyzed quantities—response amplitudesA, response distancesD, and variancesSS—are illustrated schematically inFigure 6A, and the results are presented inFigure 6B–Din terms of the mean values and standard errors for every run. In identity-selective parcels, response amplitudes$A_{fam}$to recurring patterns decreased during the first session (runs 1 to 6,p<0.05), but not in the second and third session (runs 7 to 12, runs 13 to 18,p>0.5). Response amplitudes$A_{nov}$to non-recurring patterns showed no significant change (pn.s.) in any session (Fig. 6B). In non-selective parcels, response amplitudes decreased in all sessions, consistent with general habituation. In identity-selective parcels, response distances$D_{diff}$between different recurring objects declined similarly during the first session (p<0.05), but not during subsequent sessions (p>0.6) (Fig. 6C). Also, response distances$D_{same}$between the same recurring objects did not change significantly during any session (pn.s.). In contrast, response distances$D_{nov}$between non-recurring objects declined disproportionately during the first session (p<0.05) but increased during the third session (p<0.05). Response distances$D_{novfam}$between recurring and non-recurring objects, on the other hand, did not change significantly over sessions (pn.s.).

A first conclusion is that response amplitudes and response distances are consistently larger for recurring objects (blue traces inFig. 6B, C) than for non-recurring objects (red traces). Importantly, in the very first run, response distances are comparable between different recurring objects ($D_{diff}$) and different non-recurring objects ($D_{nov}$), demonstrating that both recurring and non-recurring objects were represented comparably well. Over subsequent runs, response distances decrease far more between different non-recurring objects ($D_{nov}$) than different recurring objects ($D_{diff}$), demonstrating that a comparative advantage for*recurring*objects develops gradually (i.e., a kind of repetition enhancement). A second conclusion is that the observed development differs between identity-selective and non-selective parcels. Whereas amplitudes and distances stabilize in the former group of parcels, they habituate progressively in the latter group (both within and between sessions). Thus, the responsiveness of identity-selective parcels remains stable over sessions. A third conclusion is that response distances$D_{nov}$between different non-recurring objects become comparatively small (already during the first session), not only smaller than the distances$D_{diff}$between*different*recurring objects but even smaller than the distances$D_{same}$between the*same*recurring objects.

The results for response variances confirmed the trends observed earlier in the batch analysis of cross-validated variance ratios (Fig. 4A, B). Between-class variance$SS_{diff}$for recurring objects declined over the course of sessions (p<0.005), whereas between-class variance$SS_{novfam}$for non-recurring objects increased over the first session (p<0.005), only to decline again during the third session (p<0.05). Within-class variances$SS_{same}$and$SS_{nov}$remained largely unchanged. The close correspondence between the trends observed over runs and over batches is illustrated also in SupplementaryFigure S5. Surprisingly, non-identity-selective parcels mirrored the trends observed for identity-selective parcels in attenuated form. The fact that between- and within-class variances differ systematically suggests that even non-identity-selective parcels represent object identity to some degree.

It is natural to compare these results to the time-course of behavioral performance (fraction correct and reaction time) in our observersFig. 1C, D). The changes in the representation of*recurring*objects (between class distances$D_{diff}$and variances$SS_{diff}$) show a gradual*decrease*in the quality of representation and thus do*not*correspond to improving performance in terms of fraction correct. However, the changes in the representation of*non-recurring*objects, including the decrease of within-class distances$D_{nov}$and variances$SS_{nov}$and the increase of between-class variances$SS_{novfam}$and variance ratio$R_{novelty}$, do correspond to the rapid improvement in fraction correct over the first few runs. Thus, the neural changes over the course of learning point to diverging representations of “novel” (non-recurring) and “familiar” (recurring) objects.

### 3.4 Stability of identity and novelty representations

Relying on fixed subspacesSto analyze each data set also permitted us to assess the*stability*of neural representations over successive runs. With this in mind, we established the centroids of response classes for each run and examined the displacement of centroids between successive runs. As this calculation concerned centroid-to-centroid distances (rather than exemplar-to-exemplar distances), we could not correct for temporal auto-correlations.

The computation of centroids for particular response classes is illustrated schematically inFigure 7A. Given the centroids$C_{r-1}$and$C_{r}$for successive runsr−1andrand the average centroid$C_{aver}$over all runs, we computed absolute centroid-to-centroid distances$DC_{r}=\left\|\;C_{r}-C_{ave}\;\right\|$as well as relative centroid-to-centroid distances$\Delta DC_{r}=\left\|\;C_{r}-C_{r-1}\;\right\|$. The 16 response classes were formed by each recurring object (15 classes,$DC_{same}$and$\Delta DC_{same}$) and by the non-recurring objects (1 class,$DC_{nov}$and$\Delta DC_{nov}$). To compare the displacements expected from sampling noise, we also computed the centroid-to-centroid distances after permuting the responses in each class and regrouping them into 18 “pseudo-runs” (seeMethodsfor details).

**Fig. 7.**
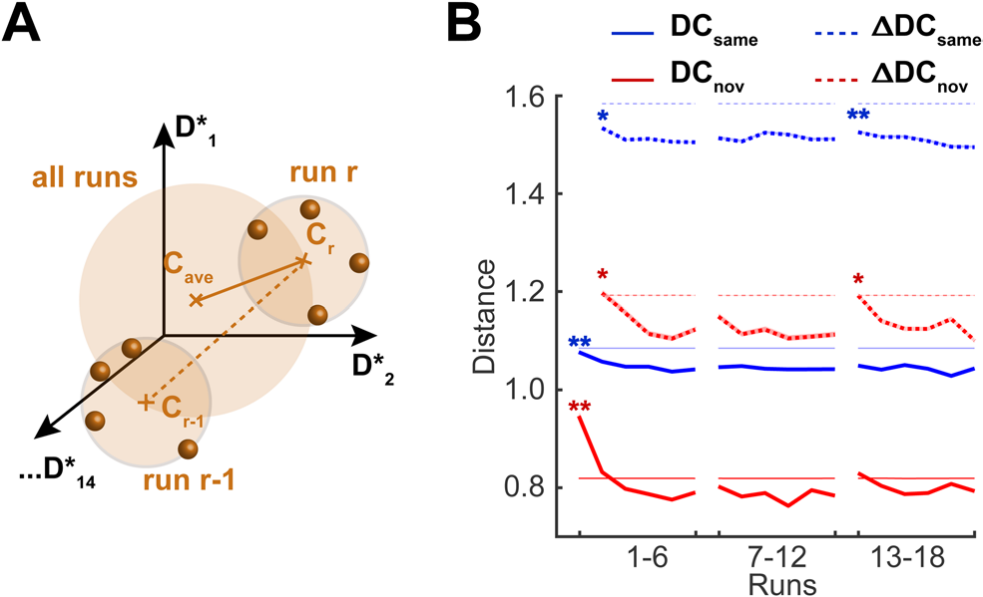
Stability of identity and novelty representations over successive sessions and runs (A) For each recurring and all non-recurring objects, we calculated the response centroids$C_{r}$for each runrand the average centroid$C_{ave}$for all runs and obtained both absolute distances$DC_{r}=\left\|\;C_{r}-C_{ave}\right\|$and relative distances$\Delta DC_{r}=\left\|\;C_{r}-C_{r-1}\right\|$. (B) Centroid-to-centroid distances (mean±S.E.M.) for all identity-selective parcels and all data sets. Distances$DC_{same}$and$\Delta DC_{same}$for the same recurring objects (blue) and distances$DC_{nov}$and$\Delta DC_{nov}$for non-recurring objects (red) are compared to the corresponding values obtained from shuffled data sets (thin, pale lines). Stars indicate a significant linear trend during a session (see text).

The results are shown inFigure 7B. For both recurring and non-recurring objects, average absolute distances$DC_{same}\left(r\right)$and$DC_{nov}\left(r\right)$diminished during the first session (runs 1 to 6,p<0.005), but remained stable during the second and third sessions (runs 7 to 12, and 13 to 18,p>0.2). Notably, absolute distances$DC_{nov}\left(r\right)$of novel objects decreased to a much lower average level. Relative distances$\Delta DC_{same}\left(r\right)$and$\Delta DC_{nov}\left(r\right)$between successive runs declined during the first session (runs 1 to 6,p<0.05), remained stable during the second session (runs 7 to 12,p>0.2), only to decline once again the last session (13 to 18,p<0.005for recurring andp<0.05for non-recurring objects). Absolute distances were far larger for recurring than for non-recurring classes, corroborating the substantial “response enhancement” already noted above. Both absolute and relative distances were slightly smaller than predicted by sampling noise (thin, pale lines,p<0.001), demonstrating that responses of true runs were distributed slightly more compactly and consistently than those of pseudo-runs. Note also that relative distances approached the values expected for fully random displacements in a 14-dimensional hypersphere—specifically, relative distancesΔDCwere approximately1.4times larger than absolute distancesDC– again underlining the dominant influence of sampling noise.

## 4 Discussion

We studied the cortical representation of synthetic visual objects over multiple days of repeated viewing, while observers learned to classify initially unfamiliar objects as “familiar.” Relying on “representational similarity analysis” (RSA), we established distances between spatiotemporal hemodynamic (BOLD) responses to exemplars of different*recurring*objects, as well as to exemplars of*non-recurring*objects. Response distances between the same and different recurring objects quantified the neural representation of object*identity*. Response distances between recurring and non-recurring objects measured the neural representation of object*novelty*. The results showed that object identity was neurally represented from the start, in the ventral occipitotemporal cortex and beyond. With growing familiarity, the quality of this neural representation remained high, but its geometry expanded to fill the available representational space. In contrast, the neural representation of non-recurring objects (which remained “novel” by definition) improved over time, but its geometry contracted and shifted to the margins of the representational space.

### 4.1 Cortical representation of object identity

To permit a fine-grained analysis of representational geometry, we generated complex and three-dimensional shapes that were highly characteristic and distinguishable and presented these shapes from various points of view and in various states of rotation (always for one complete turn) (Kakaei et al., 2021). Thus, observers had to recognize an object from all sides in order to classify it as “familiar.” Within the category of our synthetic shapes, every recurring object constituted strictly speaking an “exemplar,” with individual presentations providing different “instantiations.” However, we chose to term objects “classes” and individual presentations “exemplars,” as this terminology conforms better to RSA conventions.

The selectivity of cortical parcels for object identity was assessed in optimized 14-dimensional subspacesSof the much higher-dimensional space of multivariate responses ($O(10^{3})$dimensions). Specifically, we computed a cross-validated “classification accuracy” (Kriegeskorte, Mur, & Bandettini, 2008) and used a prevalence analysis to combine results from different conditions and observers (Allefeld et al., 2016). Essentially identical results were obtained with alternative measures such as “linear discriminability” and “variance ratio” (of between- and within-class variance;Anderson, 2001). When spatiotemporal responses to different objects are linearly discriminable, they form a neural representation of object identity. As exemplars of each object were presented from various sides, any such neural representation was by definition view-invariant. The obvious caveats are*(i)*that object rotation may have exposed the same characteristic features in many or most presentations and*(ii)*that multivariate hemodynamic responses over 9 s can only distantly reflect the neuronal activity evoked during each 2.5 s presentation. Nevertheless, hemodynamic signals exhibited significant invariance to the various modes of presentation of a given object (e.g., the initial perspective, the axis, and the sense of rotation).

In contrast to many other studies, we did not observe suppressed responses when objects were repeated (i.e., no “repetition suppression”) but rather a small enhancement of responses both with longer delays and later trial numbers (SupplementaryFig. S1). This may simply reflect the fact that the object presentations were highly variable and never repeated exactly. Recall that we designed a highly variable display such as to retain the observers’ interest over 3 successive days.

The 124 of 758 parcels that were identified as “identity-selective” on this basis were situated mostly in the ventral occipitotemporal cortex, but some parcels were also located in the parietal or frontal cortex, as illustrated inFigure 3A. The degree of selectivity exhibited a clear gradient, being stronger at the posterior pole and becoming progressively weaker in more anterior and more dorsal regions, as summarized inFigure 8A. These results are consistent with previous findings that multivariate activity distinguishing different exemplars of a particular class of objects (e.g., faces) is present in the ventral and lateral occipital cortex, on the fusiform gyrus, and in the ventral temporal cortex (Brants et al., 2016;Eger et al., 2008;Visconti di Oleggio Castello et al., 2021).

**Fig. 8.**
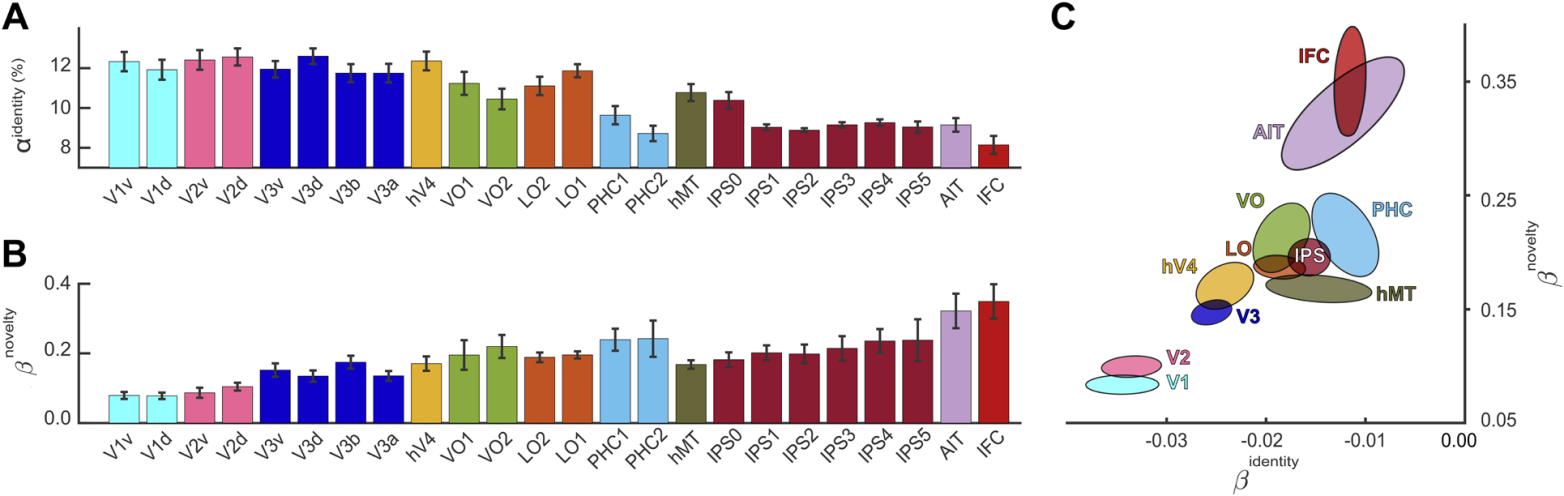
Shape identity and shape novelty representations in 26 topographical regions. (A) Identity representation as indexed by classification accuracy$\alpha^{identity}$(mean±S.E.M.). Posterior regions (V1-hV4, VO, LO) exhibit higher accuracy than more anterior or more dorsal regions (IPS, AIT, IFC). (B) Novelty representation as indexed by rate$\beta^{novelty}$of novelty gain (mean±S.E.M.). More anterior or more dorsal regions (IPS, AIT, IFC) exhibit a higher slope parameter than the posterior visual cortex (V1-hV4). (C) Comparison of identity and novelty representations as indexed by rate$\beta^{identity}$of identity loss (negative values) and rate$\beta^{novelty}$of novelty gain (positive values). Groups of regions are distinguished by color, with ellipsoids indicating mean and standard error. Note the positive correlation between novelty*gain*and identity*loss*. List of abbreviations: visual cortex (V1, V2, V3, hV4), ventral occipital cortex (VO1, VO2), lateral occipital cortex (LO1, LO2), parahippocampal cortex and fusiform gyrus (PHC), medial temporal areas (hMT), intraparietal sulcus (IPS), anterior inferior temporal cortex (AIT), and inferior frontal cortex (IFC).

In general, it is thought that progressively “higher” levels of visual processing represent progressively “larger” visual sets, beginning with image features, and widening gradually to object features, object exemplars, object categories, and finally to supercategories such as animate or inanimate objects, or objects and landscapes (Grill-Spector & Weiner, 2014). Accordingly, the discriminability of exemplars within a category is expected to diminish at more anterior locations, which correspond to “higher” levels of visual processing (Eger et al., 2008;Grill-Spector & Weiner, 2014). Moreover, it has been hypothesized that the spatial scale of neural representations increases with the level of abstraction, in the sense that exemplars are represented at smaller scales than categories (Grill-Spector & Weiner, 2014). Thus, if this trend is exacerbated in the more anterior parts of the ventral pathway, exemplar representations may become progressively less discriminable at the spatial resolution of BOLD signals.

A previous study of visual expertise for synthetic shapes (Brants et al., 2016) reported a gradual enhancement of neural representations in object-selective areas, whereas we observed a moderate decline. This difference may have been due to task design. Brants and colleagues used barely discriminable shapes and emphasized perceptual load, whereas we used highly distinguishable shapes and emphasized memory load.

We also observed identity-selectivity in frontoparietal regions that are typically associated with the dorsal visual pathway and the right frontoparietal “attention network.” This is consistent with previous findings on the presence of object- and/or face-selective representations in dorsal areas (Freud et al., 2017;Jeong & Xu, 2016;Konen & Kastner, 2008;Poirier et al., 2006;Visconti di Oleggio Castello et al., 2021). However, the interpretation of this selectivity is not straightforward. Particularly the clusters associated with the “attention network” are often found to express functional correlations with ventral visual areas in both resting and task states (Dornas & Braun, 2018;Mutlu et al., 2022;Smith et al., 2013). Thus, it seems possible that multivariate functional correlations could have propagated identity-selectivity feedforward throughout the “attention network” and beyond.

Finally, we observed pronounced identity-selectivity in the primary visual cortex (calcarine sulcus, left and right), where neuronal activity encodes basic visual features (orientation, spatial frequency, direction of movement, and so on) (Grill-Spector & Weiner, 2014;Haxby et al., 2001). It is possible that multivariate hemodynamic responses in the primary visual cortex could have reflected this visually evoked neuronal activity sufficiently well to have encoded object identity, especially as the rotation may have exposed the same low-level features in many or most presentations. Additionally, hemodynamic responses could have been driven by spatiotemporal patterns of feedback from higher areas of the visual cortex. There is some evidence to suggest that feedback can dominate the hemodynamics of the early visual cortex under continuous viewing conditions (as used here) (Blake & Braun, 2009).

### 4.2 Cortical representation of novel object shapes

We also investigated the representation of “novel” object shapes that were encountered only once (and never recurred). Note that “novelty” is here not meant to imply “surprise” for the observer in the sense of a violation of expectations (e.g.,Uddin, 2015). Rather, it simply denotes the more heterogeneous class of*non-recurring*objects (with 360 exemplars, each from a different object), as distinct from the 15 more homogeneous classes of*recurring*objects (with approximately 200 exemplars each, all from the same object). As mentioned, “novelty” was measured in terms of the linear discriminability of hemodynamic responses to non-recurring and recurring objects in 14-dimensional subspacesS, more specifically, by comparing pairwise response distances between classes (recurring and non-recurring) and within classes (either recurring or non-recurring).

All 124 “identity-selective” parcels were also “novelty-selective,” in the sense that hemodynamic responses discriminated non-recurring and recurring objects to some degree, as illustrated inFigure 5A. As discriminative subspaces were optimized for recurring objects—that were generated in the same way as non-recurring objects—some degree of discriminability was to be expected. Moreover, as non-recurring objects were more numerous (360 objects) than recurring objects (15 objects), some discriminability was expected purely by chance, particularly in a 14-dimensional space. However, as discussed further below, the linear discriminability of non-recurring objects increased over successive runs and sessions, mirroring observers’ improving ability to classify objects as “novel” or “familiar.” Because of this dynamic aspect, we quantified the novelty-selectivity of cortical parcels in terms of an “improvement rate,”$\beta^{novelty}$(Fig. 4). Interestingly, there was an anterior-posterior gradient in that novelty-selectivity was more pronounced in more frontal, parietal, and anterior temporal areas than more posterior temporal and occipital areas, as summarized inFigure 8B. In other words, the representational disparity between familiar object shapes and novel objects shapes tended to be larger in the higher-level (more anterior) visual cortex than in the lower-level (more posterior) cortex, suggesting that learning effects were more pronounced.

### 4.3 Representational changes with learning

As representational changes with learning were the main objective of our study, we addressed this issue with several complementary approaches. First, we divided our observations from 18 runs into five successive “batches” and established the neural representation of both “identity” and “novelty” separately for each batch with cross-validated statistics, while aggregating over all identity-selective parcels (Fig. 4B). Second, to assess changes in individual parcels, we performed a regressional analysis of the same cross-validated data and obtained “rates” of representational changes for every identity-selective parcel (Fig. 4C). Third, we adopted stable discriminative subspacesSand sacrificed cross-validation in order to analyze representational geometry over individual runs (Fig. 6). All three approaches yielded comparable results.

Already in the first run and the first batch, without time for plasticity or learning, the neural representations of identity were*maximally*differentiated (Figs. 4Aand6D; SupplementaryFig. S5). This initial identity representation was most pronounced in known object processing areas, including the ventral occipitotemporal cortex and early visual cortex. Apparently, pre-existing representations based on life-long experience were sufficient to immediately provide a view-independent representation of synthetic shapes, which we had designed to be highly characteristic and discriminable. In contrast, neural representations of novelty were*minimally*differentiated in the first run and the first batch. As there was no systematic difference between recurring and non-recurring objects (and without time for plasticity), any residual initial discriminability of novelty must be attributed to chance.

Over subsequent runs and batches, the neural representation of object identity remained pronounced, but its quality declined steadily over time (Figs. 4Aand6D; SupplementaryFig. S5). Some decline in BOLD activity is not untypical for learning studies over multiple days and is commonly ascribed to repetition suppression, sparsification of responses, and/or diminishing attention or effort (e.g.,Poldrack, 2000). However, while our results are consistent with such a scenario in non-identity-selective parcels, they do not support a general decline of activity in identity-selective parcels, as the response amplitudes and distances in these parcels declined only initially and subsequently remained stable (Fig. 6B, C).

In contrast, the neural representation of object novelty improved substantially over subsequent runs and batches. The time course was similar in both analyses (batch-by-batch and run-by-run), with the steepest improvement occurring over the first few runs (Figs. 4Aand6D; SupplementaryFig. S5). However, the detailed results revealed that this “improvement” (in discriminating non-recurring and recurring objects) actually reflected a deterioration in the representation of non-recurring objects (i.e., diminishing response distances,Fig. 6C).

In absolute terms, response amplitudes and distances were already larger for recurring objects and smaller for non-recurring objects during the first run and the difference increased over the next few runs (Fig. 6B, C). Apparently, recurring objects benefited from a “repetition enhancement,” as the only immediate and systematic difference between recurring and non-recurring objects was the frequency of recurrence. Interestingly, this enhancement was comparable for “structured” and “unstructured” sequences, even though the repetition latencies were quite different (SupplementaryFig. S1B, C). Accordingly, we hypothesize that the enhancement was not merely a passive effect but rather a consequence of task relevance and cognitive engagement (SupplementaryFig. S1B, C).

As mentioned, the rates of change of identity and novelty representations differed systematically between cortical regions (Fig. 8C). Intriguingly, the rates of novelty*gain*and identity*loss*varied inversely over the cortical hierarchy: in early visual areas (V1, V2, V3, hV4), identity declined rapidly, whereas novelty grew slowly. At the opposite end, in the inferior frontal cortex (IFC) and anterior ventral temporal cortex (AIT), identity declined slowly, but novelty grew rapidly. In the higher visual cortex (VO, LO), both rates were intermediate.

**Fig. 9.**
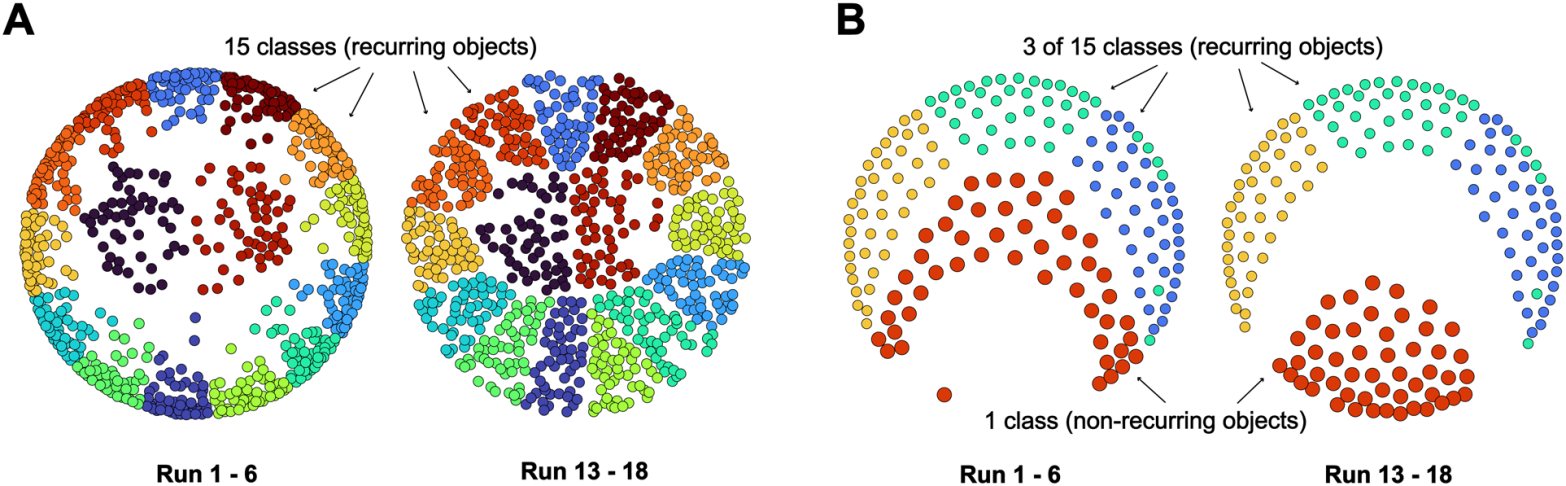
Changes in the geometry of shape identity and novelty representations, visualized with multi-dimensional scaling. Symbols (colored circles) represent neural response patterns in a 14-dimensional spaceS. Symbols are positioned such that pairwise distances reflect pairwise distances inS. Response classes are distinguished by color and are represented by 50 randomly selected responses each. (A) Fifteen response classes to recurring objects in the first session (left, run 1–6) and the third session (right, run 13–18). Note that*recurring*response classes expand with learning to fill the available space. (The regions occupied by classes depend on the selected responses. “Inside” and “outside” classes can exchange positions). (B) Three response classes to recurring objects and one response class to non-recurring objects (larger symbols), in the first session (left, run 1–6) and the third session (right, run 13–18). Note that the*non-recurring*response class contracts with learning and shifts to the margins of the available space. Class positions are similar for other triplets of recurring classes.

It is informative to visualize the observed representational changes in two dimensions (Fig. 9), while approximately preserving the*relative*pairwise distances in the discriminative subspacesS. This visualization makes clear that the neural representation of recurring objects expands between the beginning and the end of the experiment, filling the available representational space (Fig. 9A). The expansion explains our observation that the linear discriminability of object classes degrades but remains high. In contrast, the neural representation of non-recurring objects contracts between the beginning and the end of the experiment while also shifting to the margins of representational space, which explains why the linear discriminability of non-recurring objects improved over time (Fig. 9B). These two opposite developments may reflect both cognitive engagement and repetition frequency: representations may expand for objects that observers attempt to memorize and/or that recur frequently, but contract for objects that observers learn to ignore and/or that are rare.

In addition to relative changes in representational geometry indexed by linear discriminability, we established absolute changes in representational geometry, indexed by distances between response centroids in successive runs (seeFig. 7). The results were dominated by sampling noise, and the displacement of centroids was comparable to random jumps in a hypersphere while maintaining a given distance from its center. However, both absolute and relative centroid distances were slightly (and significantly) smaller than predicted by sampling noise, indicating that the representations were slightly more consistent and compact. The most interesting result of this analysis was that centroid distances were approximately30%smaller for non-recurring than for recurring objects, highlighting again the representational disparity noted above.

### 4.4 Behavioral and cognitive changes with learning

The behavioral changes over three sessions of viewing sequences of objects included both increased classification performance (“familiar” or “novel”) and decreased reaction times. Both behavioral measures changed rapidly during the first three runs of the first session and more slowly during the second and third sessions (Fig. 1). As described elsewhere (Kakaei et al., 2021), the classification of a particular object typically changed from (mostly) “novel” to (mostly) “familiar” at one identifiable point in time during the sessions, which we termed “onset of familiarity.” This objective observation was consistent with the subjective reports of observers that they memorized all three-dimensional shapes one by one, such that every object became recognizable from all sides. Some observers also mentioned having assigned linguistic labels to individual recurring objects. After the three sessions, all observers were “familiar” with all recurring objects and could pick them out from an array of distractor objects.

Only some of these behavioral changes have obvious counterparts in the neural changes discussed above. First, the decrease of reaction times from under 2 s to under 1 s implies that observers spend less time actively evaluating the stimulus and more time passively observing it. However, the neural response of identity-selective parcels does not mirror this trend, as both response amplitudes and response differences stabilize after the first few runs (Fig. 6B, C). In the rest of the brain (non-identity-selective parcels), the neural responses do show a progressive decrease, but any attribution would be speculative.

Second, the increase in objective performance and in subjective “familiarity” was not mirrored directly in neural responses to recurring objects, as multivariate responses were sufficiently rich to identify such objects from the very start. However, multivariate responses were dispersed over the three sessions such as to fill more of the available space (see above). This growing response diversity is a plausible correlate of memory consolidation, that is, the formation of stable long-term memories in visually responsive cortical areas. When such memories are consolidated, one would expect that increased connectivity would enhance pattern completion over additional levels of representation, rendering network activity more complex (e.g.,Steinberg & Sompolinsky, 2022). It is worth noting that this development was observed for both types of presentation sequences (“structured” and “unstructured”), suggesting that neural consolidation was due to task relevance and not merely to repetition latency.

Third, the increase in objective performance was mirrored indirectly in neural responses to*non-recurring*objects. Whereas these responses were initially comparable to recurring responses, they contracted over three sessions into a smaller part of the available space, thus becoming more stereotypical. As this part was comparably distant from all recurring responses, it lay at the margins of the representational space. The time course of classification performance corresponded best to this particular development in neural representations. Accordingly, this development was a plausible*indirect*correlate of memory consolidation, in the sense that visually responsive areas grew*less responsive*to other objects that failed to match the newly formed long-term memories.

## 5 Conclusion

We analyzed the cortical representation of visual objects in the multivariate hemodynamic responses of 758 brain parcels. For each parcel, we used linear discriminant analysis to map the O($10^{3}$)-dimensional responses into a lower-dimensional subspace that optimally discriminated the 15 stimulus classes (recurring objects). Optimal subspaces captured a large part of the correlated variance and overlapped substantially with the principal components of the responses. Typically, 2/3 of the principal component variance discriminated between stimulus classes (and thus coincided with the optimal subspace), while the remaining 1/3 was shared between stimulus classes. Our analyses revealed where and how the cortical representations of visual objects changed as visual expertise was being acquired and consolidated by the observers.

Our results were broadly consistent with other recent studies of visual expertise, which have highlighted the roles of three pathways or networks (Kravitz et al., 2011,^2013^), an occipitotemporal pathway (“ventral pathway”), an occipitoparietal pathway (“dorsal pathway”), and a right frontoparietal network (“attention system”). Several studies linked behavioral performance to enhanced activity and/or representation in the frontoparietal network (Duyck et al., 2021;Poirier et al., 2006;Visconti di Oleggio Castello et al., 2021), as well as in the more anterior parts of the occipitotemporal pathway and the more dorsal parts of the occipitoparietal pathway (Christophel et al., 2017).

Due to our focus on object shape, our results do not speak directly to the modulation of cortical responses by expectation, such as “expectation suppression” or “surprise signalling” (Barron et al., 2016;Bell et al., 2016;Mayrhauser et al., 2014; Vinken et al., 2018). Moreover, in our paradigm, object presentations were never repeated exactly and every object presentation contained elements of surprise, as neither the object, nor the point of view, nor the direction of rotation could be anticipated by observers.

The most robust representations of object shape for both recurring objects (“identity”) and non-recurring objects (“novelty”) were observed in the ventral occipitotemporal cortex, at the intermediate levels of the shape processing hierarchy (Grill-Spector & Weiner, 2014;Perry & Fallah, 2014). Additionally, we found representations of object shape in “dorsal stream” cortical areas, consistent with the view that these areas encode goal- and task-related object features (Perry & Fallah, 2014).

The most novel aspect of our findings was changes in the geometry of cortical representations as visual expertise for recurring objects was being acquired and consolidated. In relative terms, distances between response classes decreased, and/or distances within classes increased, while observers repeatedly viewed and became familiar with the corresponding stimulus classes. This modest*decline*in stimulus encoding was however associated with an expansion (or*diversification*) in the distribution of responses within classes, so that responses of all classes taken together scattered more uniformly over the available representational space. Changes in cortical representations were quite different for stimuli that appeared only once and that observers did not attempt to memorize (non-recurring objects). Here, again in relative terms, distances between classes (non-recurring and recurring) increased and/or distances within classes (non-recurring) decreased. This steep*growth*in class encoding was associated with a substantial contraction (or*stereotypisation*) in the distribution of responses, in the sense that responses to non-recurring objects shifted to the margin of the available representational space.

We conclude that hemodynamic responses to novel object shapes immediately represent the differences between these shapes, even prior to learning, presumably reflecting life-long prior experience. When object shapes grow familiar with learning, hemodynamic responses to the same shapes become more diverse, whereas responses to different shapes remain comparably dissimilar from each other. Responses to control objects that are always novel develop quite differently in that they become less diverse relative to each other, but also more dissimilar from responses to familiar objects.

## Data and Code Availability

Direct linear discriminant analysis and prevalence inference is available on github.com/cognitive-biology/DLDA. MR data will be made available upon request.

## Author Contributions

Ehsan Kakaei: Conceptualization, data curation, formal analysis, visualization, and writing of the original draft. Jochen Braun: Conceptualization, linear algebra, formal analysis, supervision, and reviewing & editing.

## Supplementary Material

Supplementary Material

## AppendiX

**Appendix Table A1:** List of identity-selective parcels and their anatomical region.

AAL region	Parcel	MNI			α (%)	β	$p^{⋆}$	$p^{⋆}$	$p^{⋆}$	Topog.
	No.	x	y	z	Identity	Novelty	Struct.	Unstruct.	Both	Assign.
Precentral	14	-51	7	36	8.4	0.33	0.006	$10^{-5}$	$10^{-5}$	-
*Superior frontal*	*56*	25	-9	64	7.9	0.18	n.s.	n.s.	$4\times 10^{-5}$	-
Inferior frontal (opercular)	143	38	11	31	7.5	0.27	$5\times 10^{-5}$	$5\times 10^{-3}$	0.02	IFC
	146	52	10	22	9	0.44	0.05	n.s.	$10^{-5}$	IFC
Inferior frontal (triangular)	163	51	23	20	7.9	0.34	$10^{-5}$	$10^{-5}$	$10^{-5}$	IFC
Parahippocampal	325	26	-38	-9	7.5	0.05	0.007	$2\times 10^{-4}$	$10^{-5}$	-
Calcarine	331	-2	-91	-1	16.9	0.01	$10^{-5}$	$10^{-5}$	$10^{-5}$	V1v
	333	-2	-94	-4	10.2	-0.01	$10^{-5}$	$5\times 10^{-3}$	$10^{-5}$	V1v
	335	-4	-86	7	12.6	0.11	$10^{-5}$	$10^{-5}$	$10^{-5}$	V1d
	336	-5	-99	-8	12.2	0.03	$10^{-5}$	$10^{-5}$	$10^{-5}$	V1v
	337	-12	-99	-5	13.8	0.10	$10^{-5}$	$10^{-5}$	$10^{-5}$	V1d
	338	-7	-75	10	8.7	0.08	$10^{-5}$	$10^{-5}$	$10^{-5}$	-
	342	17	-83	11	9.8	0.07	$10^{-5}$	$10^{-5}$	$10^{-5}$	-
	344	9	-85	6	13.8	0.10	$10^{-5}$	$10^{-5}$	$10^{-5}$	V1v
	345	15	-91	1	14.3	0.09	$10^{-5}$	$10^{-5}$	$10^{-5}$	V1d
	347	18	-99	-1	12.1	0.07	$10^{-5}$	$10^{-5}$	$10^{-5}$	V1d
	348	11	-72	11	9.1	0.06	n.s.	$10^{-5}$	$2\times 10^{-4}$	-
Cuneus	350	-7	-85	26	10.1	0.07	$10^{-5}$	$10^{-5}$	$10^{-5}$	-
	352	0	-94	25	9.1	0.05	$10^{-5}$	$10^{-5}$	$10^{-5}$	V2d
	354	-2	-79	22	8.6	0.07	0.003	$10^{-5}$	$10^{-5}$	-
	355	-4	-92	25	11.9	0.09	n.s.	$10^{-5}$	0.02	V2d
	356	12	-91	18	13	0.15	$10^{-5}$	$10^{-5}$	$10^{-5}$	V2d
	357	15	-97	10	13.8	0.08	$10^{-5}$	$10^{-5}$	$10^{-5}$	V2d
Lingual	363	-12	-65	-5	9.5	0.10	$10^{-5}$	$10^{-5}$	$10^{-5}$	V3v
	364	-15	-95	-16	10.9	0.02	$10^{-4}$	$10^{-5}$	$10^{-5}$	V2v
	367	-29	-89	-16	11.5	0.07	$2\times 10^{-5}$	$10^{-5}$	$10^{-5}$	hV4
	368	-17	-85	-12	14.9	0.26	$10^{-5}$	$10^{-5}$	$10^{-5}$	V2v
	370	-22	-65	-5	9.7	0.16	$10^{-5}$	$10^{-5}$	$10^{-5}$	VO2
	371	-12	-79	-8	13.9	0.12	$10^{-5}$	$10^{-5}$	$10^{-5}$	V2v
	372	-6	-74	2	12	0.11	$10^{-5}$	$10^{-5}$	$10^{-5}$	V1v
	373	16	-81	-7	14.1	0.11	$10^{-5}$	$10^{-5}$	$10^{-5}$	V3v
	375	11	-72	-4	12.5	0.04	$10^{-5}$	$10^{-5}$	$10^{-5}$	V2v
	377	21	-58	-3	9.3	0.24	$10^{-5}$	$10^{-5}$	$10^{-5}$	-
	378	13	-52	2	7.6	0.05	n.s.	$10^{-5}$	$2\times 10^{-4}$	-
	379	16	-88	-10	13.4	0.14	$10^{-5}$	$10^{-5}$	$10^{-5}$	V2v
	380	27	-91	-16	9.2	0.06	n.s.	0.01	0.01	-
	381	17	-98	-10	9.3	0.04	$10^{-5}$	n.s.	0.005	V1v
	383	14	-56	-6	7.9	0.05	$2\times 10^{-5}$	0.01	$10^{-5}$	-
Occipital (superior)	384	-18	-84	25	10.6	0.08	$10^{-5}$	$10^{-5}$	$10^{-5}$	V3a
	385	-16	-85	41	9.8	0.12	$10^{-5}$	$10^{-5}$	$10^{-5}$	IPS0
	386	-18	-69	29	7.7	0.04	n.s.	$10^{-5}$	0.005	-
	387	-16	-95	23	13.5	0.12	$10^{-5}$	$10^{-5}$	$10^{-5}$	V3a
	388	-22	-75	34	9.1	0.21	$10^{-5}$	$10^{-5}$	$10^{-5}$	IPS1
	389	-11	-95	9	12.9	0.05	$10^{-5}$	$10^{-5}$	$10^{-5}$	V2d
	390	22	-90	24	13.6	0.10	$10^{-5}$	$10^{-5}$	$10^{-5}$	V3a
	391	21	-98	14	13.8	0.11	$10^{-5}$	$10^{-5}$	$10^{-5}$	V2d
	392	27	-85	40	9.2	0.12	$10^{-5}$	$10^{-5}$	$10^{-5}$	IPS0
	*393*	*25*	-67	33	8	0.18	n.s.	n.s.	0.005	-
	394	24	-75	21	8.7	0.11	$10^{-5}$	$10^{-5}$	$10^{-5}$	-
	395	23	-79	33	10	0.12	$10^{-5}$	$10^{-5}$	$10^{-5}$	-
	396	29	-70	43	9.2	0.21	0.03	$10^{-5}$	$10^{-5}$	IPS1
Occipital (middle)	397	-28	-77	27	10.1	0.23	$10^{-5}$	$10^{-5}$	$10^{-5}$	IPS0
	398	-28	-72	34	9.1	0.32	n.s.	$10^{-5}$	$10^{-5}$	IPS1
	400	-38	-86	4	12	0.15	$10^{-5}$	$10^{-5}$	$10^{-5}$	LO2
	402	-33	-87	19	12	0.24	$10^{-5}$	$10^{-5}$	$10^{-5}$	V3b
	*403*	-30	-78	2	7.7	0.05	n.s.	n.s.	0.02	-
	404	-27	-94	1	12.7	0.15	$10^{-5}$	$10^{-5}$	$10^{-5}$	V3d
	405	-16	-100	1	14	0.13	$10^{-5}$	$10^{-5}$	$10^{-5}$	V2d
	406	-40	-75	15	8.9	0.14	$10^{-5}$	$10^{-5}$	$10^{-5}$	-
	407	-27	-83	14	11.3	0.19	$10^{-5}$	$10^{-5}$	$10^{-5}$	IPS0
	408	-24	-93	13	13.6	0.19	$10^{-5}$	$10^{-5}$	$10^{-5}$	V3d
	410	-38	-83	23	9.9	0.21	$10^{-5}$	$10^{-5}$	$10^{-5}$	-
	412	-46	-77	5	10.1	0.14	$10^{-5}$	$10^{-5}$	$10^{-5}$	hMT
	413	33	-88	7	13.2	0.23	$10^{-5}$	$10^{-5}$	$10^{-5}$	LO1
	414	33	-96	4	10.7	0.17	$10^{-5}$	$10^{-5}$	$10^{-5}$	V3d
	415	39	-81	14	10.6	0.19	$10^{-5}$	$10^{-5}$	$10^{-5}$	V3b
	416	44	-78	5	10.9	0.22	$10^{-5}$	$10^{-5}$	$10^{-5}$	LO2
	418	32	-86	23	11.9	0.22	$10^{-5}$	$10^{-5}$	$10^{-5}$	V3b
	420	34	-69	32	8.5	0.34	n.s.	$10^{-5}$	0.02	-
	421	32	-76	27	10	0.28	$10^{-5}$	$10^{-4}$	$10^{-5}$	IPS0
Occipital (inferior)	423	-50	-68	-14	9.4	0.22	$10^{-5}$	$10^{-5}$	$10^{-5}$	-
	424	-31	-83	-8	12.6	0.22	$10^{-5}$	$10^{-5}$	$10^{-5}$	-
	425	-22	-95	-9	12.3	0.11	$10^{-5}$	$10^{-5}$	$10^{-5}$	-
	426	-42	-73	-8	11.6	0.21	$10^{-5}$	$10^{-5}$	$10^{-5}$	-
	428	36	-85	-7	13.2	0.21	$10^{-5}$	$10^{-5}$	$10^{-5}$	-
	430	42	-73	-9	11.4	0.25	$10^{-5}$	$10^{-5}$	$10^{-5}$	-
Fusiform	432	-27	-71	-11	12.2	0.25	$10^{-5}$	$10^{-5}$	$10^{-5}$	VO2
	435	-33	-77	-17	12.4	0.15	$10^{-5}$	$10^{-5}$	$10^{-5}$	hV4
	436	-31	-53	-13	8.8	0.26	$10^{-5}$	n.s.	$10^{-5}$	PHC1
	438	-41	-56	-17	8.9	0.26	n.s.	$10^{-5}$	0.01	-
	440	-36	-63	-16	9.6	0.25	0.001	$10^{-5}$	$10^{-5}$	-
	442	28	-74	-11	12.8	0.31	$10^{-5}$	$10^{-5}$	$10^{-5}$	hV4
	443	36	-71	-16	10.8	0.25	0.02	$10^{-5}$	$10^{-5}$	hV4
	447	29	-47	-14	8.3	0.31	0.002	$10^{-5}$	$10^{-5}$	PHC2
	450	41	-48	-20	8.4	0.30	0.006	$10^{-5}$	0.02	-
	452	28	-59	-12	9.7	0.35	$10^{-5}$	$10^{-5}$	$10^{-5}$	VO2
Postcentral	476	60	-18	37	9.2	0.34	$10^{-5}$	$10^{-5}$	$10^{-5}$	-
	478	42	-31	49	8.5	0.25	$10^{-5}$	0.02	$10^{-5}$	-
	484	30	-40	61	8.5	0.20	$10^{-5}$	$10^{-5}$	$10^{-5}$	-
Parietal (superior)	494	-26	-61	61	9.1	0.09	$10^{-5}$	$10^{-5}$	$10^{-5}$	-
	495	-27	-53	68	8	0.09	0.02	0.05	$2\times 10^{-5}$	-
	497	-21	-67	47	9.3	0.19	$10^{-5}$	$10^{-5}$	$10^{-5}$	IPS1
	498	-15	-69	50	8.5	0.12	0.01	$10^{-5}$	$10^{-5}$	IPS2
	499	-28	-69	50	8.9	0.29	0.001	$3\times 10^{-5}$	$10^{-5}$	-
	501	-17	-79	50	8.7	0.11	$10^{-4}$	$3\times 10^{-5}$	$10^{-5}$	IPS1
	502	30	-60	64	8.8	0.18	0.002	$5\times 10^{-4}$	$10^{-5}$	IPS3
	504	34	-62	57	9.3	0.30	$5\times 10^{-5}$	0.003	$10^{-5}$	-
	506	33	-50	58	9.8	0.34	$10^{-5}$	$10^{-5}$	$10^{-5}$	-
	507	21	-57	74	8.1	0.09	n.s.	$10^{-5}$	$5\times 10^{-4}$	-
	509	31	-73	53	8.6	0.13	n.s.	$10^{-5}$	0.001	-
	*510*	21	-74	55	8.8	0.14	0.06	0.07	$2\times 10^{-5}$	IPS1
	511	20	-65	54	9.2	0.17	$10^{-5}$	$10^{-5}$	$10^{-5}$	IPS2
Parietal (inferior)	514	-45	-30	42	8.6	0.24	0.002	$10^{-5}$	$10^{-5}$	-
	516	-32	-75	44	8	0.19	0.01	$5\times 10^{-4}$	$10^{-5}$	-
	521	-39	-47	42	8.6	0.17	n.s.	$10^{-4}$	0.005	-
	522	-32	-46	50	8.5	0.12	$10^{-5}$	$10^{-5}$	$10^{-5}$	-
	523	-31	-53	46	8.4	0.18	$10^{-5}$	n.s.	$2\times 10^{-5}$	-
	527	46	-39	50	8.6	0.35	n.s.	$10^{-5}$	0.001	-
	529	37	-49	46	8.7	0.34	0.06	0.003	$2\times 10^{-5}$	-
	530	35	-44	51	9.2	0.29	$5\times 10^{-4}$	$10^{-5}$	$10^{-5}$	-
Supramarginal	536	-61	-28	34	8.4	0.24	0.003	$10^{-5}$	$10^{-5}$	-
	539	44	-34	41	8.7	0.20	0.001	0.001	$10^{-5}$	-
	542	63	-24	37	8.5	0.22	$10^{-5}$	n.s.	$10^{-4}$	-
Angular	557	34	-60	44	8.7	0.38	n.s.	$10^{-5}$	0.005	-
Precuneus	561	-5	-77	53	7.9	0.06	$10^{-5}$	$2\times 10^{-5}$	$10^{-5}$	-
	573	-9	-71	54	8.1	0.08	$10^{-5}$	0.003	$10^{-5}$	-
	576	14	-71	45	7.9	0.12	$10^{-5}$	$10^{-5}$	$10^{-5}$	-
Temporal (middle)	678	-45	-67	11	8.9	0.12	$10^{-5}$	n.s.	$10^{-4}$	-
	685	-49	-62	0	9.3	0.19	$10^{-5}$	$10^{-5}$	$10^{-5}$	-
	701	52	-59	3	8.9	0.20	$10^{-5}$	$10^{-5}$	$10^{-5}$	-
	717	49	-69	4	10.5	0.18	$10^{-5}$	$10^{-5}$	$10^{-5}$	-
Temporal (inferior)	728	-54	-58	-11	8.5	0.25	$2\times 10^{-4}$	$10^{-5}$	$10^{-5}$	AIT
	732	-45	-52	-13	9.3	0.30	$10^{-5}$	$10^{-5}$	$10^{-5}$	AIT
	755	46	-53	-11	9.7	0.42	$10^{-5}$	$10^{-5}$	$10^{-5}$	AIT

Parcel ID, geometrical centroid x/y/z in MNI coordinates, average classification accuracyα, average novelty rateβ, corrected significance$p^{⋆}$in structured or unstructured conditions (n=8), corrected significance$p^{⋆}$in both conditions (n=16), and topographical assignment, if any.
