# Supplementary material for: Gradual change of cortical representations with growing visual expertise for synthetic shapes
Source: Imaging Neurosci (Camb). 2024 Aug 6;2:imag-2-00255. doi: 10.1162/imag_a_00255 (PMC12272194; doi:10.1162/imag_a_00255)
Supplement: Supplementary Material [file imag_a_00255-supp.pdf]

Supplementary information: Gradual change of cortical representations with growing visual expertise for synthetic shapes.

Ehsan Kakaei,[1,2,3*] Jochen Braun,[2,3]

[1]European Structural and Investment Funds Graduate School on Analysis, Imaging, and Modelling of Neuronal and Inflammatory Processes, Otto-von-Guericke University, 39120 Magdeburg, Germany

[2]Institute of Biology, Otto-von-Guericke University, 39120 Magdeburg, Germany

[2]Center for Behavioral Brain Sciences, Otto-von-Guericke University, 39120 Magdeburg, Germany

*Correspondence: ehsankakaei91@gmail.com

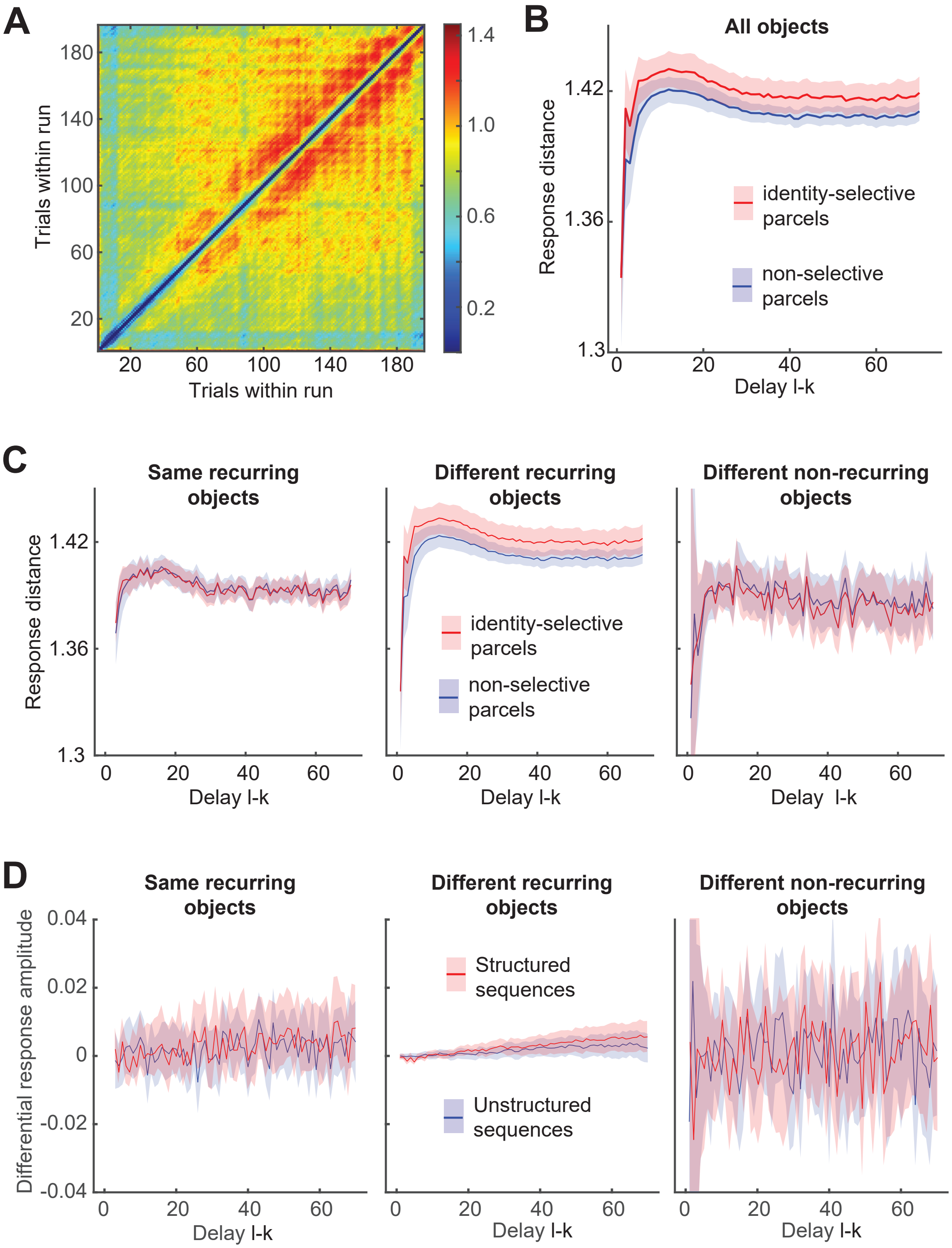

Figure S1: Responses distances and amplitudes as a function of delay. Pairwise response distances $d_{w,u,r}(k,l)$ between trials k and l in parcel w, run r and subject u were averaged over subjects and runs to obtain $D_w(k,l) = \langle d_{w,u,r}(k,l) \rangle_{u,r}$. Similarly, differential response amplitudes $a_{w,u,r}(l) - a_{w,u,r}(k)$ between earlier trials k and later trials l in parcel w, run r and subject u were averaged over subjects and runs to obtain $A_w(k,l)$. **A)** Pattern of response distances for all pairs of trials k, l, average $\langle D_w(k,l) \rangle_w$ over all parcels. **B**) Pairwise response distances $D(k,l)$ as a function of delay, $l-k$, average, and SEM (solid trace and shading) over identity-selective parcels (red) and non-identity-selective parcels (blue). **C)** Pairwise response distances for different types of objects, same recurring, different recurring, and different non-recurring. **D)** Differential response amplitudes $A(k,l)$ for different types of objects and different types of presentation sequences, structured sequences (red) and unstructured sequences (blue).

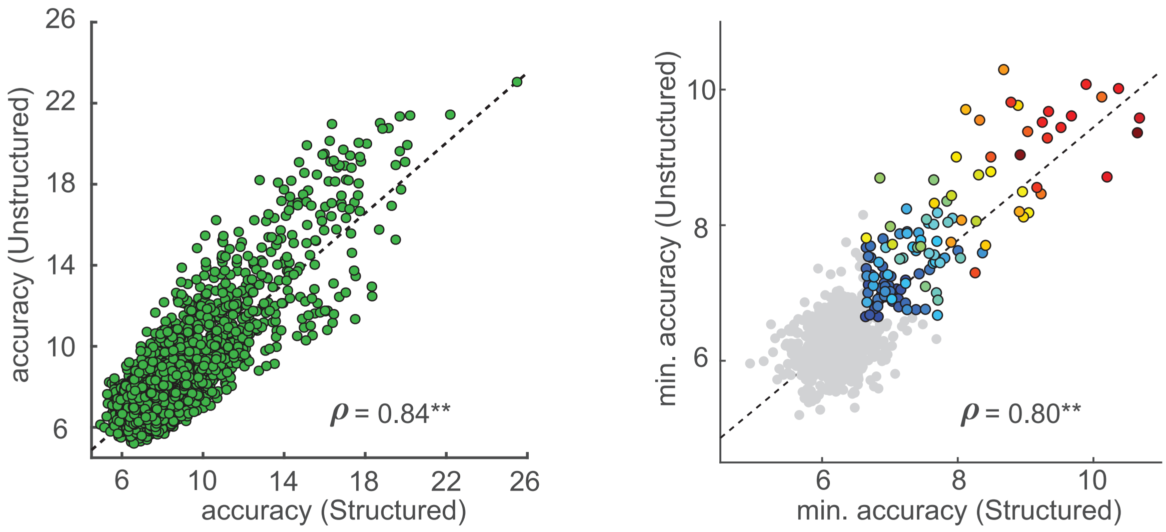

Figure S2: Classification accuracy $a_{w,u}$ for all observers u and parcels w, as observed with structured or with unstructured presentation sequences. **A)** Accuracy values observed with structured and unstructured sequences are correlated ($\rho = 0.842$, $p < 0.001$). **B)** Minimal accuracy values of all parcels w, obtained by combining observers. Values from structured and unstructured sequences are correlated ($\rho = 0.802$, $p < 0.001$). Colors indicate the combined accuracy of parcels categorized as identity-selective.

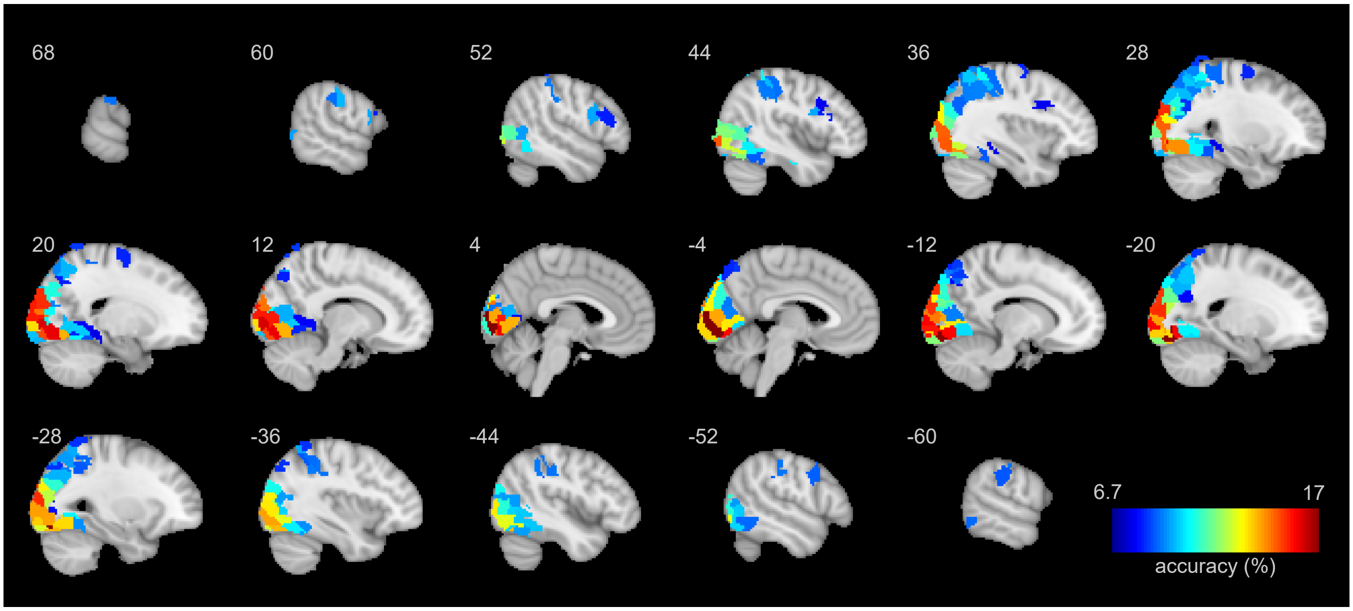

Figure S3: Cortical discriminability of object ‘identity’. Color scale indicates the average classification accuracy of identity-selective parcels, ranging from chance level (6.67%) to maximum (15%). Sagittal slices (8 mm thickness) range from $X = -60$ to $X = +68$ (MNI).

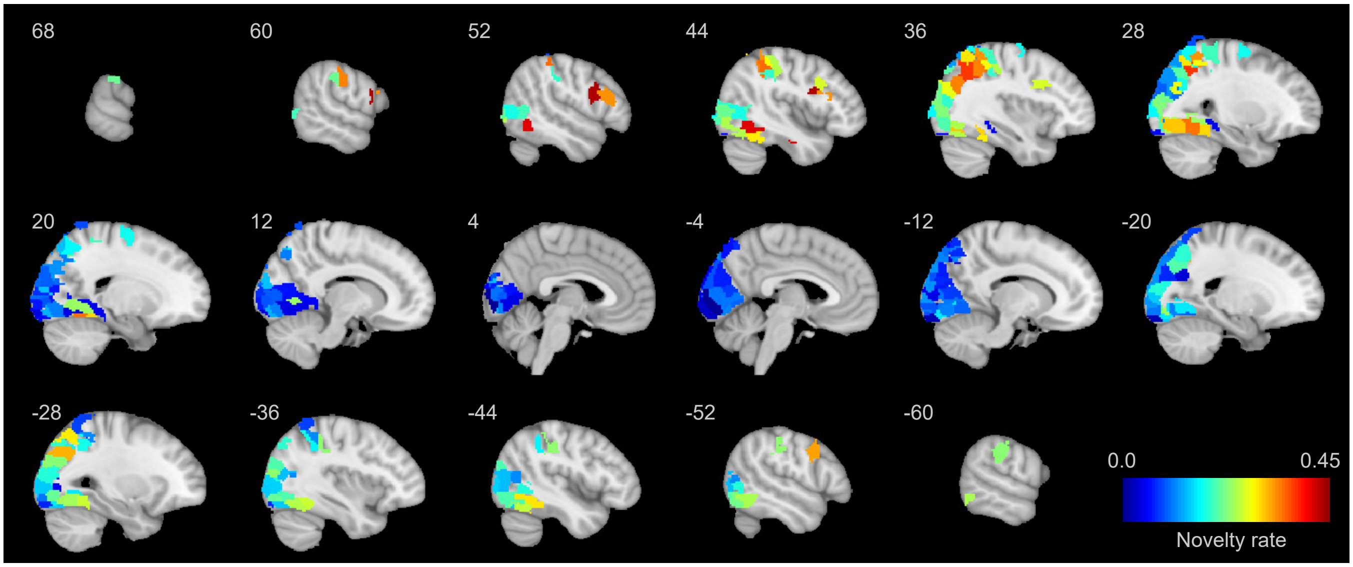

Figure S4: Cortical discriminability of object 'novelty'. Color scale indicates the average rate $\beta^{novelty}$ of increase in the variance ratio $F^{novelty}$, ranging from a minimal value of -0.01 to a maximal value of 0.44. Sagittal slices (8 mm thickness) range from $X = -60$ to $X = +68$ (MNI).

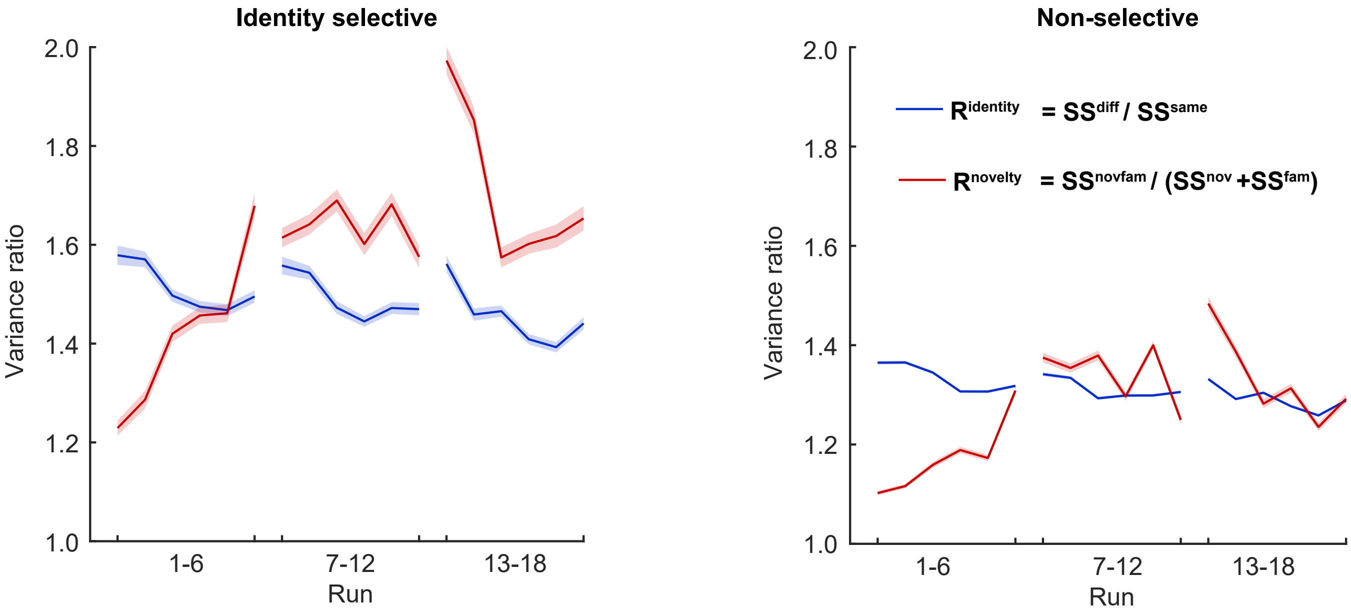

Figure S5: Development of variance fractions (mean $\pm S.E.M$) over the course of experiment (18 runs in 3 session). Variance ratios $R^{novelty}$ (red) and $R^{identity}$ (blue) were computed for every run, and averaged separately for identity-selective (left) and non-identity-selective parcels (right).

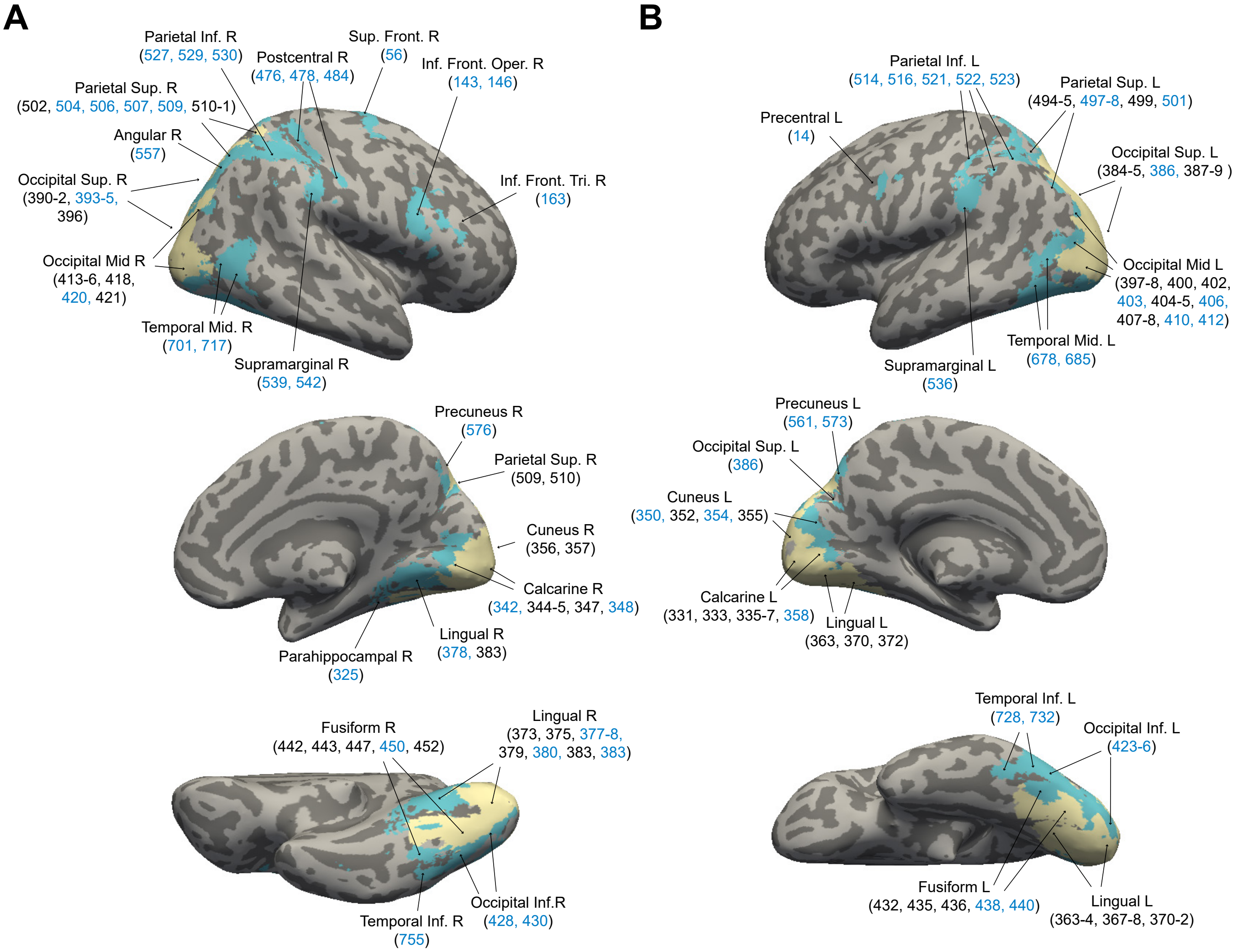

Figure S6: Overview of cortical parcels selective for identity. Topographically assigned regions in the visual cortex (in beige) are distinguished from other cortical regions (in blue). **A** Right hemisphere, lateral (top), medial (middle), and ventral views (bottom). **B** Left hemisphere, lateral (top), medial (middle), and ventral views (bottom).
